# Visual Identification of *Trichosporon asahii,* a Gut Yeast Associated with Obesity, Using an Enzymatic NIR Fluorescent Probe

**DOI:** 10.1021/acs.analchem.2c01691

**Published:** 2022-08-03

**Authors:** Lei Feng, Ying Deng, Shufan Song, Yanqiu Sun, Jingnan Cui, Xiaochi Ma, Lingling Jin, Yan Wang, Tony D. James, Chao Wang

**Affiliations:** † Second Affiliated Hospital, Dalian Medical University, Dalian 116023, China; ‡ Dalian Key Laboratory of Metabolic Target Characterization and Traditional Chinese Medicine Intervention, College of Pharmacy, College of Integrative Medicine, Dalian Medical University, Dalian 116044, China; § School of Chemistry and Chemical Engineering, Henan Normal University, Xinxiang 453007, China; ∥ State Key Laboratory of Fine Chemicals, Dalian University of Technology, Dalian 116024, China; ⊥ Department of Chemistry, University of Bath, Bath BA2 7AY, U.K.

## Abstract

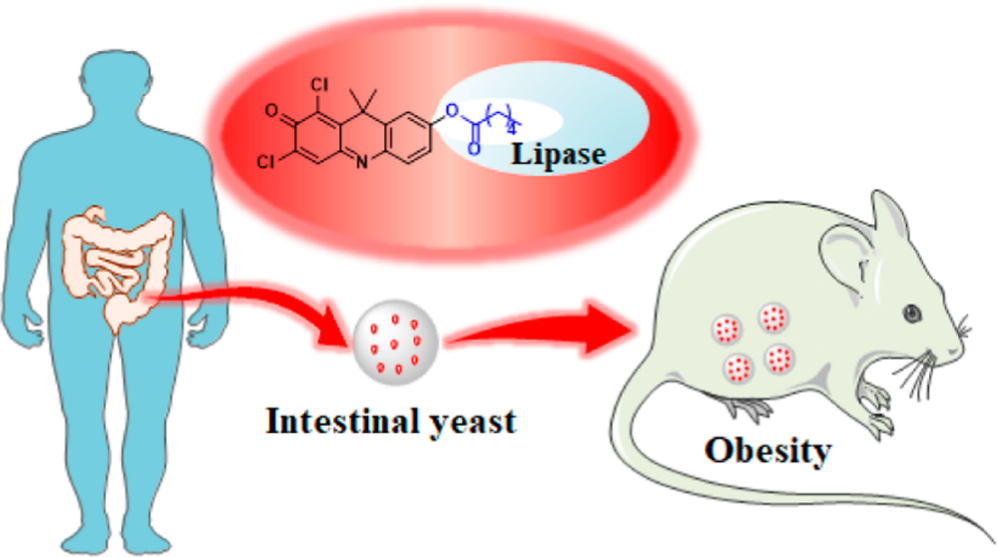

Lipase found in the gut microbiota participates in the digestion and absorption of dietary fats. As such, the gut microbiota is involved in the regulation of the host metabolism, affecting the levels of lipids and free fatty acids, ultimately resulting in obesity. In this study, an enzymatic activatable near-infrared fluorescent probe, **DDAO-C6**, was developed for visually sensing endogenous lipase from gut microbes. Using **DDAO-C6**, a cultivated intestinal yeast strain was rapidly identified from human feces that exhibited high lipase expression and was identified as *Trichosporon asahii* Y2. We then determined that the colonization of the gut of mice with *T. asahii* Y2 increased lipase activity in the digestive tract and promoted obesity and hyperlipidemia when the mice were fed high fat diets. Above all, the present research resulted in a fluorescence visualization tool for the functional investigation of gut microbiota associated with obesity and disorders of lipid metabolism.

Gut microbes are part of a complex community inhabiting the gastrointestinal tract, which consists of bacteria, eukaryotic cells, and fungi, as well as a small population of viruses.^1,2^ Current research points to the important role played by the gut microbiota in the health and wellbeing of the host organism.^3−8^ The existence of a symbiotic relationship has also been suggested between the gut microbiota and the host, and as such the link between metabolic functions and gut microbiota has attracted significant attention.^9^ For example, research has clearly indicated a direct role of intestinal bacteria in the regulation of human metabolism, which can result in obesity and metabolic syndrome. Recent investigations have revealed a close relationship between the gut microbiota and the metabolism of lipids in the gut of the host, affecting the levels of lipids and free fatty acids.^10,11^ As such, gut microorganisms with active lipase are key factors involved in the development of metabolic syndrome and obesity.^12^

Lipase (E.C. 3.1.1.3) is a serine hydrolase secreted by gut microbiota that catalyzes the hydrolysis of various lipids, such as cholesteryl esters, triglycerides, diglycerides, monoglycerides, phospholipids, and ceramides.^13^ There is strong evidence that lipase greatly contributes to the digestion and absorption of dietary fats,^14^ by breaking down oil in the food source into small molecules such as glycerol and fatty acids which the body can absorb.^15^

The role of gut bacteria in human health and the roles of gut bacteria containing active lipase in the development of obesity, metabolic syndrome, and regulating the metabolism are known.^12^ However, the role of gut fungi remains unclear, and the function of gut fungi in the health of the host requires more research. Therefore, as important constituents of the gut microbiota, gut fungi with active lipase need to be identified and their roles in obesity need to be investigated, as such an efficient sensing method for gut fungal lipase is urgently needed.

Due to the rapid development of fluorescence-based technologies, a significant number of fluorescent probes have been developed for the visual sensing and activity assays of various biological enzymes in different species, such as mammals, bacteria, and fungi.^16−24^ For the activity assay of lipase, colorimetric assays based on *para*-nitrophenol (pNP) acyl esters, fluorogenic esterase substrates based on coumarin, fluorescein, rhodamine, naphthalimide, and resorufin, and self-assembled micelles have been developed.^25−33^ However, no fluorescent probe has been developed for sensing the lipase of gut microbiota, in order to explore the function of gut microbiota in obesity and metabolic syndrome.

With this research, a near-infrared (NIR) fluorophore 7-hydroxy-9*H*-(1,3-dichloro-9,9-dimethylacridin-2-one) (**DDAO**) as a reporter was linked with a series of acyl esters including medium- and long-chain fatty acids. Using **DDAO** hexanoate (**DDAO-C6**) as a fluorescent probe, the intestinal fungus (*Trichosporon asahii* Y2) that exhibits high lipase expression was identified from human feces. Furthermore, *T. asahii* Y2 was shown to promote nutritional obesity in mice. Therefore, a reliable and sensitive detection tool for lipase activity has been developed to be suitable for the investigation of gut microbiota and the early diagnosis and monitoring of metabolic syndromes such as dyslipidemia, obesity, and hyperglycemia.

## Experimental Section

### Materials and Methods

Chemical reagents (e.g., petroleum ether, ethyl acetate, and dichloromethane) for the synthesis of the fluorescent probe were produced by Tianjin Kemio Chemical Reagent Co., Ltd (China). The constituents for culture medium preparation such as tryptone, agar, glucose, and penicillin (10 KU/mL)/streptomycin (10 mg/mL) were purchased from Dalian Meilun Biotechnology Co., Ltd (China). Lipase, aminopeptidase N (APN), tyrosinase (PPO), laccase, pyruvate oxidase (PC), xanthine oxidase (XO), bovine serum albumin (BSA), and human albumin (HSA) were purchased from Sigma-Aldrich (MERCK). CYP450 enzymes (CYP3A4, CYP1A2, and CYP2B6) were purchased from Corning Incorporated Life Sciences. Commercial kits (IL-1β, TNF-α, and ApoB) were purchased from Elabscience Biotechnology Co., Ltd (China). A commercial kit to assay lipase activity was obtained from Nanjing jiancheng Bioengineering Institute (China). Kits for lipid determination (T-CHO, TG, and LDL-C) were purchased from Nanjing jiancheng Bioengineering Institute (China). Hepatic tissue sections stained by H&E and oil red O were prepared by Wuhan Servicebio Biotechnology Co., Ltd (China).

NMR spectra of the synthesized compounds were acquired using a Bruker-600 with tetramethylsilane (TMS) as the internal standard (Bruker, USA). High-resolution mass spectroscopy (HRMS) was measured using a X500R QTOF-MS (SCIEX, USA). high-performance liquid chromatography (HPLC) analysis was performed on a Dionex UltiMate 3000 equipped with a diode array detection (DAD) detector and a C18 silica gel column (Thermo Scientific, USA). A constant temperature incubator shaker (HZQ-C) was purchased from Harbin Donglian Electronic Technology Development Co., LTD (China). Fluorescence images of cells were obtained using a confocal laser scanning microscope was manufactured by Leica (Germany). The fluorescence spectra and fluorescence intensity were recorded using a Synergy H1 microplate reader (BioTek, USA). Fluorescence imaging of agar plates and 96-well plates stained by **DDAO-C6** were performed using an Amersham Typhoon RGB (GE, USA).

### Synthesis of Fluorescent Probes for Lipase Activity

Using 7-hydroxy-9*H*-(1,3-dichloro-9,9-dimethylacridin-2-one) (**DDAO**) as the fluorophore, a series of medium and long-chain fatty acids as the recognition groups of lipases were attached to the hydroxyl moiety of **DDAO**, affording a series of **DDAO** esters. The synthetic routes (Schemes S1 and S2), procedures, and spectroscopic data of these **DDAO** esters are available in the Supporting Information.

### Enzymatic Hydrolysis of DDAO-C6 Mediated by Lipase

The enzymatic hydrolysis of **DDAO-C6** mediated by lipase was performed in saline. 200 μL saline containing lipase (50 μg/mL) and **DDAO-C6** (10 μM) were shaken at constant temperature (37 °C) for 30 min. Then, 100 μL acetonitrile was used to terminate the enzymatic hydrolysis before removal of the denatured protein by centrifugation (20,000*g*, 10 min). The supernatant was subjected to a microplate reader for the measurement of the fluorescence spectra (λ_ex_ 600/λ_em_ 658 nm).

In addition, enzymatic hydrolysis of **DDAO-C6** (10 μM) was monitored by fluorescence intensity in the presence of lipase at different concentrations (0, 2, 4, 6, 8, 12, 20, 25, 30, 40, and 50 μg/mL) when incubated at 37 °C for 30 min. A linear relationship between the fluorescence intensities and lipase concentrations was then obtained. Similarly, a series of enzymatic hydrolyses of **DDAO-C6** (10 μM) in the presence of lipase (50 μg/mL) were performed with different incubation times (0, 5, 10, 15, 20, 25, 30, 35, 40, and 45 min) at 37 °C.

Selectivity for the hydrolysis of **DDAO-C6** by lipase was determined by the co-incubation of **DDAO-C6** (37 °C, 30 min) with various biological enzymes CYP3A4, CYP2B6, CYP1A2, HSA, BSA, and APN (50 μg/mL). Interference toward the fluorescence intensity of **DDAO-C6** was evaluated in the presence of various species, including ROS [H_2_O_2_, *tert*-butyl hydroperoxide (TBHP)], ions (Mn^2+^, Ca^2+^, Mg^2+^, Ni^2+^, Zn^2+^, Sn^2+^, Fe^2+^, Ba^2+^, Cu^2+^, Na^+^, K^+^, CO_3_^2–^, and SO_4_^2–^, 200 μM), and amino acids (Ser, Glu, Try, Tyr, Gly, Cys, Arg, Lys, and Gln, 10 μM).

HPLC analysis was performed using a Dionex UltiMate 3000 equipped with a DAD detector and a C18 silica gel column (4.6 × 260 mm, 5 μm). The mobile phase was set as CH_3_CN–H_2_O (0.1% TFA), with a gradient change of CH_3_CN (50–100%, 30 min, flow rate 0.8 mL/min). The detection wavelengths were set to be 220, 350, 400, 450, and 500 nm, and the chromatograms at 450 nm were used to prepare the final images.

### Identification of Intestinal Fungi with Active Lipase from Human Feces

Fresh human stools were dispersed in sterile water and coated on to a potato agar plate, which was cultured at 32 °C for about 5 days until the development of obvious fungal colonies. The potato agar plate contained penicillin (100 U/mL)/streptomycin (0.1 mg/mL), to inhibit intestinal bacteria. Then, **DDAO-C6** (100 μM) was sprayed on these colonies and incubated at 32 °C for 2 h. The plates were then imaged using an Amersham Typhoon RGB, and the fluorescence images recorded (λ_ex_ = 635 nm, λ_em_ = 670 ± 15 nm). Then, the colonies exhibiting fluorescence emission were purified and identified by the intergenic internal transcribed spacer 1 (ITS1) region sequencing using paired universal primers ITS1 (TCCGTAGGTGAACCTGCGG)/ITS2(GCTGCGTTCTTCATCGATGC), respectively.

### Fluorescence Imaging of *T. asahii* Y2 Cells by DDAO-C6

*T. asahii* Y2 was cultured in the potato medium to obtain enough cells with OD_600_ values at 1.0–1.5. The medium was removed by centrifugation (4000 rpm, 5 min). The fungal cells were washed two times using sterile saline water (1 mL) and suspended into sterile saline water (1 mL). Then, **DDAO-C6** (50 μM) was added into the fungus suspension for incubation at 32 °C (6 h). For the inhibitory experiments, orlistat (100 μM) was added into the fungal suspension together with the fluorescent probe. After incubation, the clear fungal cells were prepared by a washing procedure using sterile water and centrifugation (4000 rpm). The fungal cells were resuspended in 30 μL sterile water, which were then dropped onto a glass slide and the fluorescence images recorded using confocal laser scanning microscopy (λ_ex_ = 633 nm, λ_em_ = 645–690 nm).

### Mice Experiments

The animal experiments were approved by and performed following the guidelines of the ethics committee for animal care of the Health Sector of Dalian Medical University (approval no.: AEE19047). C57BL/6J male mice (6-week-old) were housed in pairs in SPF conditions and in a controlled environment (room temperature of 23 ± 2 °C, 12 h daylight cycle) with free access to sterile food (irradiated) and sterile water. A set of 40 mice were divided into 5 groups of 8 mice. The mice were fed a normal chow diet or a high-fat diet [60% fat and 20% carbohydrates (kcal/100 g)]. One group of high fat diet (HFD)-fed mice was treated with an oral administration of daily prepared fresh *T. asahii* Y2 at a dose of 10^8^ cfu/0.2 mL. Control groups were treated with an equivalent volume of saline or pasteurized *T. asahii* Y2 at a dose of 10^8^ cfu/0.2 mL. The body weights of the mice were measured every week. The fresh feces were collected and frozen at −80 °C weekly.

## Results and Discussion

### Development of a Fluorescent Probe to Assay Lipase Activity

**Figure 1:**
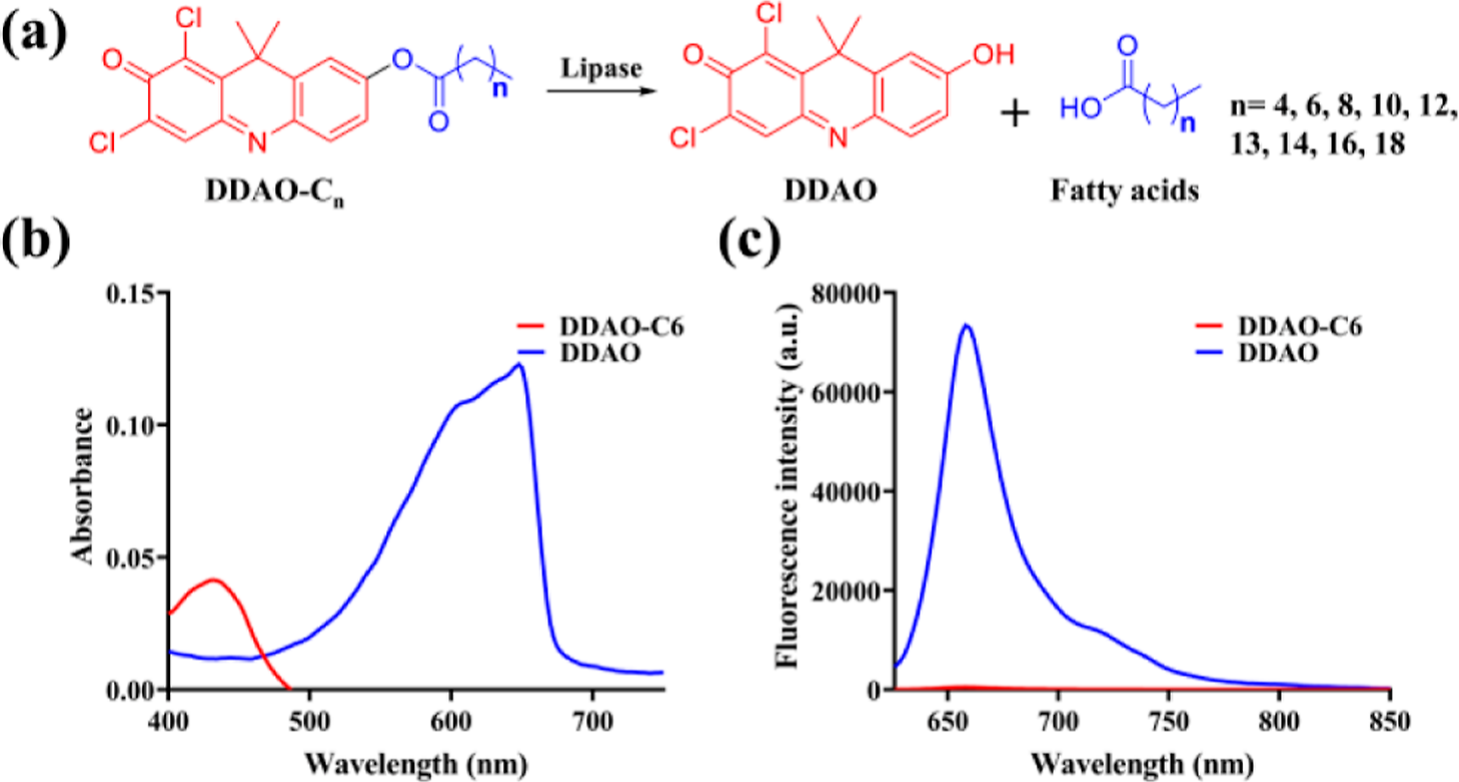
Development of a fluorescent probe to monitor lipase. (a) Illustration for the hydrolysis of the designed fluorescent probe by lipase. (b) Absorbance spectra of **DDAO-C6** and **DDAO**. (c) Fluorescence spectra of **DDAO-C6** and **DDAO**.

Lipase as a serine hydrolase catalyzes the hydrolysis of lipids (e.g., triglycerides, diglycerides, monoglycerides, and phospholipids) through the cleavage of an ester bond. In the digestive tract, lipase involved in the digestion of dietary lipids, which mainly contain long-chain fatty acids. In fact, lipase displayed the hydrase activity toward various substrates possessing long-chain (>C12) or medium chain (C6–C12) fatty acids. Therefore, **DDAO** as a NIR fluorophore with a high relative quantum yield (Φ = 0.39)^34^ was linked as esters to a series of medium and long-chain fatty acids (C6–C20), which were designed to be the substrates of lipase (Figure 1a). The fluorescent probes and hydrolyzed product **DDAO** exhibit distinct absorption and fluorescence spectra. **DDAO** exhibited an absorbance band at 600 nm, and a strong fluorescence emission at 658 nm (Figure 1b,c). Conversely, **DDAO-C6** exhibited no fluorescence at 658 nm under the same conditions. Thus, the distinct photophysical properties of **DDAO** and fatty acid derivatives led to the proposed off-on NIR fluorescent lipase probes.

**Figure 2:**
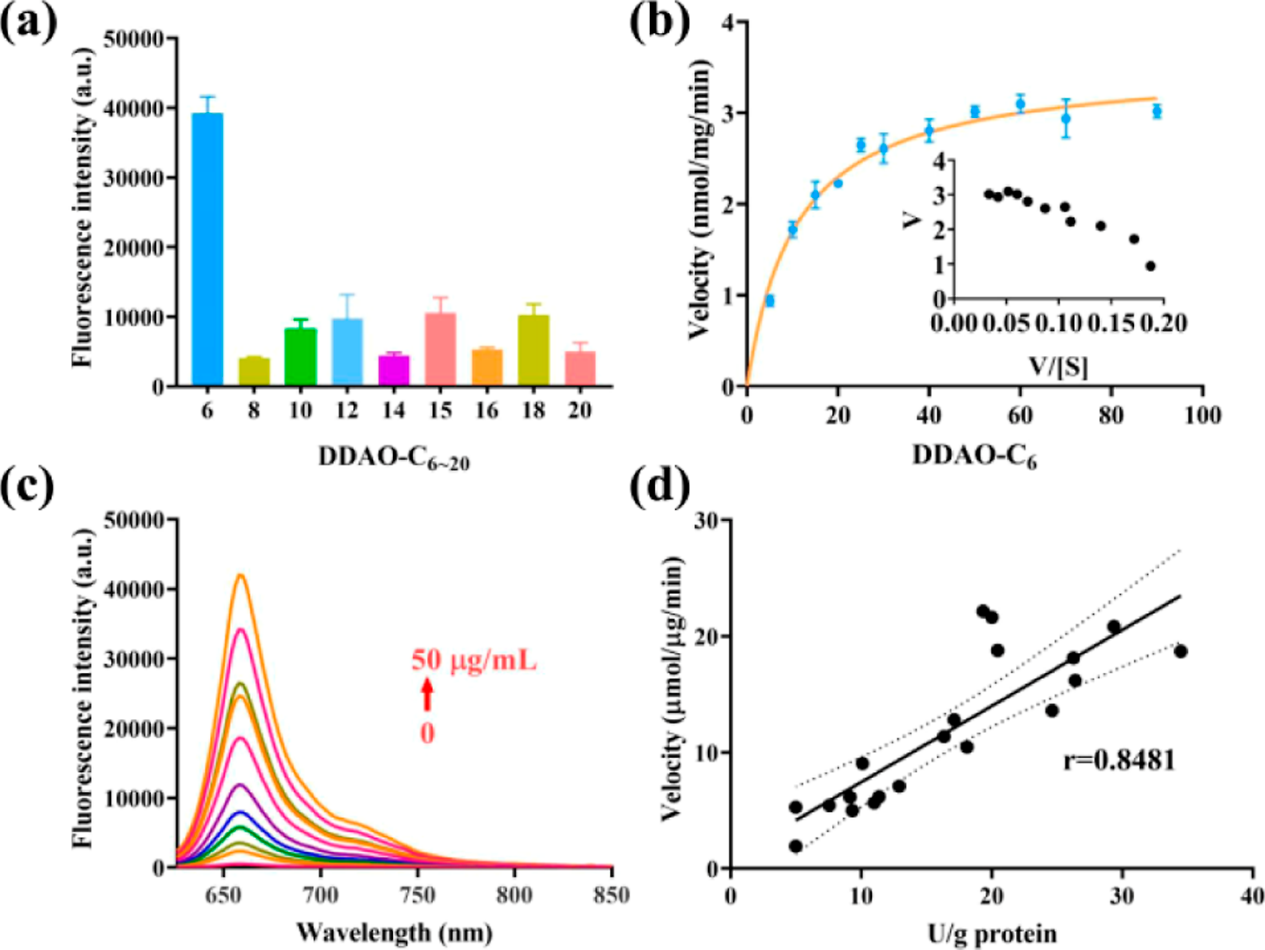
(a) Fluorescence responses of probes toward lipase. (b) Kinetics for the hydrolysis of **DDAO-C6** mediated by lipase. (c) Fluorescence behavior of **DDAO-C6** toward lipase with different activities. (d) Determination of lipase in human feces using **DDAO-C6** and commercial kit. *N* = 20.

Using these **DDAO** esters as the substrates of lipase, the enzymatic hydrolysis was evaluated using the fluorescence responses. As shown in Figure 2a, the hydrolysis of **DDAO-C6** exhibited the strongest fluorescence emission at 658 nm, indicating the best catalytic efficiency. A strong fluorescence response is required to reduce background interference within the samples evaluated.

In addition, the rapid and intense fluorescence responses of **DDAO-C6** toward lipase are a key factor for the establishment of a high-throughput assay for lipase activity in vitro. Based on HPLC analysis, the hydrolysis of **DDAO-C6** and the production of **DDAO** mediated by lipase were confirmed (Figure S1). Furthermore, the kinetics for the enzymatic hydrolysis of **DDAO-C6** mediated by lipase was investigated. Typical Michaelis–Menten kinetics were observed, with *K*_m_ = 10.82 μM and *V*_max_ = 3.539 nmol/min/mg (Figure 2b). As such, **DDAO-C6** displayed good affinity toward lipase and could be used as a fluorescent probe for the detection of lipase.

Subsequently, the fluorescence behavior of **DDAO-C6** in the presence of lipase was evaluated. The increased production of **DDAO** generated by lipase hydrolysis at different concentrations was evaluated using fluorescence spectroscopy (Figure 2c). The fluorescence intensity at 658 nm exhibited an excellent linear relationship with the lipase activity (Figure S2). Thus, it was possible to assay the activity of lipase using **DDAO-C6**. Similarly, the enzymatic hydrolysis for **DDAO-C6** with lipase was investigated. With increasing time (0–45 min), the fluorescence intensity at 658 nm increased, and a good linear relationship was observed (Figure S3). Significantly, no interference was observed for various species (e.g., bioactive proteins, amino acids, and ions) for the fluorescence responses of **DDAO** toward lipase (Figures S5 and S6). These results confirm that **DDAO-C6** could be used for the real-time detection of lipase activity under complex reaction conditions.

Lipase found in the gut is mainly responsible for the digestion and absorption of lipids from foods. Therefore, it is essential to determine the lipase activity of individuals, to facilitate the diagnosis, prevention, and therapy of diseases associated with lipid metabolism (i.e., obesity and hyperlipidemia). As such, **DDAO-C6** was used to detect lipase from individual human feces. We also determined the lipase activity in the human feces using a commercial kit. Significant differences were observed for the lipase activity of the individual feces, and a satisfactory correlation coefficient (*r* = 0.8481) was observed between the two measurement methods (Figure 2d). Therefore, **DDAO-C6** can act as a fluorescent probe suitable for the bioassay of lipase activity, as well as monitoring the digestion of lipids.

### Identification of Intestinal Fungi with Lipase Expression

It is well known that digestive lipase plays an important role in lipid metabolism. In addition to pancreatic lipase, gut microbiota is an alternative source of digestive lipase. However, there are many microbes in the human gut, making it very hard to identify an individual lipase active fungus and evaluate its role in the metabolism of lipids. Therefore, using our fluorescent probe for lipase activity, we cultivated intestinal fungus with lipase expression to explore the biological function of intestinal fungi and fungal lipase.

**Figure 3:**
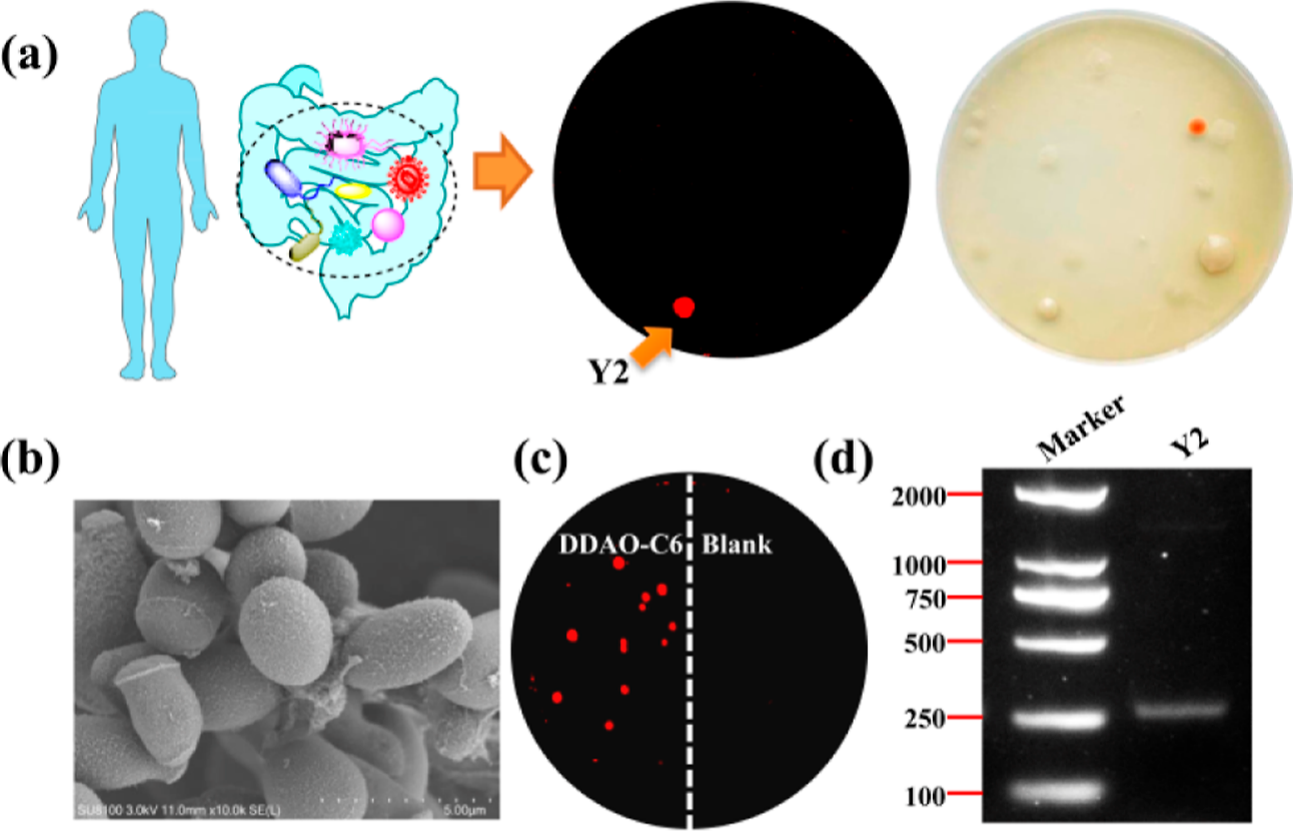
Visual identification of human intestinal fungi with high lipase activity from human feces. (a) Cultivation of intestinal fungi from human feces under the guidance of visual sensing of lipase by **DDAO-C6**. (b) SEM image of cultivated intestinal fungus Y2. (c) Fluorescence image of Y2 colonies on an agar plate. (d) Determination of lipase expression in *T. asahii* Y2 using RT-PCR.

In the present study, fresh human feces were extracted using sterile water and coated on to a potato agar medium containing penicillin/streptomycin suitable for the cultivation of intestinal fungi. After cultivation, fungal colonies were observed on the agar plate and exhibited distinct morphology (e.g., color, size, and surface) (Figure 3a). Then, to identify fungi with lipase activity before purification and gene sequencing, fluorescent probe **DDAO-C6** was dropped on to these colonies to detect endogenous lipase. The plate was then imaged, and the fluorescent colonies can be observed on the plate, indicating the existence of active lipase. Fluorescent fungal colonies were then purified, and the potential lipase active intestinal fungus was identified to be *T. asahii* Y2 by the 18S rDNA sequence.^35,36^ As purified yeast, the morphology of *T. asahii* Y2 was observed as ovoid, with no flagella using scanning electron microscopy (SEM, Figure 3b). Subsequently, the purified *T. asahii* Y2 was cultivated on a potato agar medium for lipase sensing. As shown in Figure 3c, a red fluorescence signal was observed for colonies incubated with **DDAO-C6**, suggesting the existence of active lipase. Furthermore, an RT-PCR experiment was performed to detect the existence of the mRNA corresponding to lipase. On the gel, a clear band indicated the existence of mRNA, confirming the expression of lipase in *T. asahii* Y2 (Figure 3d). Therefore, the visual sensing of endogenous lipase by **DDAO-C6** could be used to guide the rapid identification and cultivation of lipase active fungal strains from intestinal microbes, which is a potential technique for the biological investigation of intestinal microbes.

### Visual Sensing of Lipase in *T. asahii* Y2 by DDAO-C6

**Figure 4:**
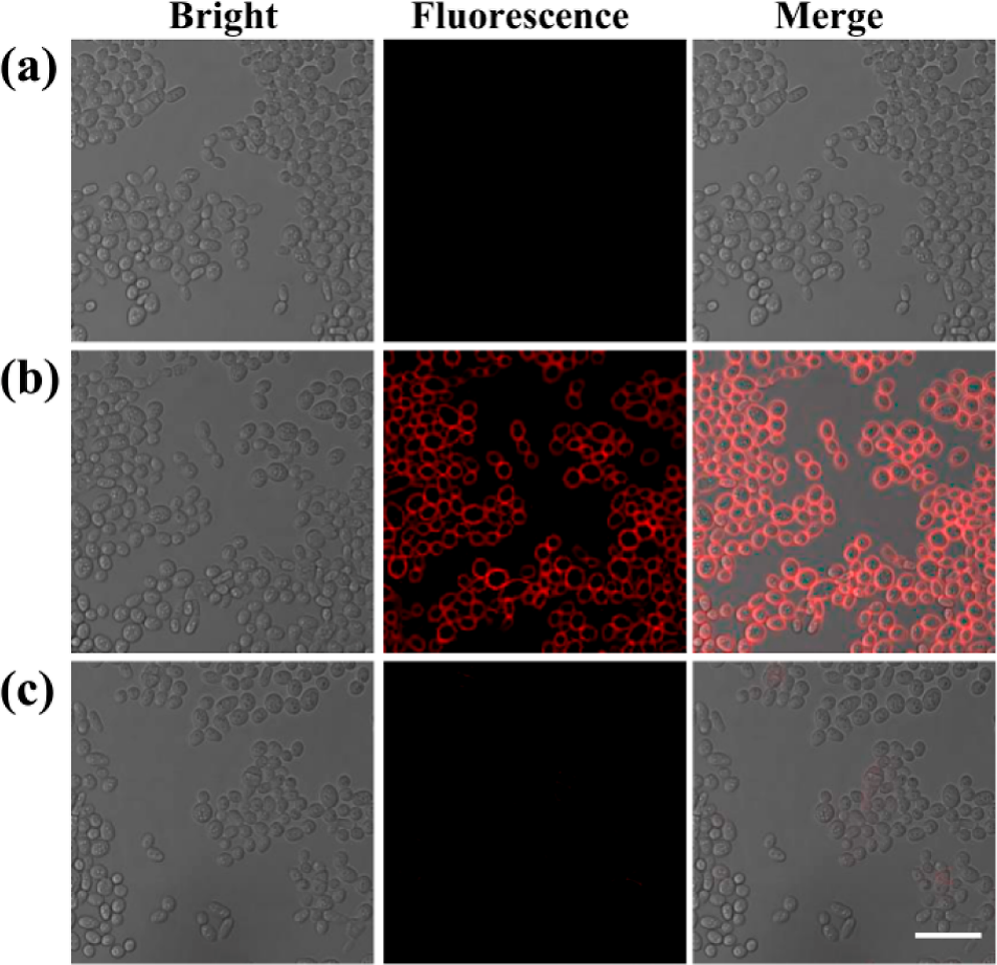
Fluorescence imaging of *T. asahii* Y2. (a) Blank group. (b) **DDAO-C6**. (c) **DDAO-C6** and orlistat. Scale bar 20 μm.

We then set out to evaluate the metabolism of **DDAO-C6** by lipase using *T. asahii* Y2 in a liquid medium. When the medium was extracted and analyzed using HPLC, the chromatograms indicated the production of **DDAO** as the hydrolysis metabolite of **DDAO-C6** (Figure S7). We then imaged the fungal cells using fluorescence confocal microscopy. As can be seen in Figure 4, compared with the blank group, a strong fluorescence signal appears for the cells incubated with **DDAO-C6**. On the other hand, fungal cells co-incubated with **DDAO-C6** and orlistat (a clinical lipase inhibitor) exhibited no fluorescence signal. Therefore **DDAO-C6** could be hydrolyzed by *T. asahii* Y2 and as such can be used to image *T. asahii* Y2 through the visual sensing of lipase activity.

Fluorescence imaging of *T. asahii* Y2 using **DDAO-C6** was then performed in the culture medium. Using **DDAO-C6**, the fluorescence images of *T. asahii* Y2 at different concentrations were measured. In comparison with the blank group, a fluorescence signal could be observed for *T. asahii* Y2 at 1.7 × 10^4^ cells/mL by the naked eye, which suggested the sensitive and convenient detection of *T. asahii* Y2 by **DDAO-C6** (Figure S8). Orlistat as a lipase inhibitor could interfere with the hydrolysis of **DDAO-C6** by *T. asahii* Y2, resulting in weak fluorescence. As shown in Figure S9, the fluorescence intensity exhibited lipase activity that correlated with the orlistat concentration. When *T. asahii* Y2 was cultivated for different times (0–48 h), the lipase activity at the various growth stages of *T. asahii* Y2 was evaluated using **DDAO-C6**.

The fluorescence intensity as a function of the cultivation time indicated strong lipase activity at the stationary stage of *T. asahii* Y2 (approximate 48 h) (Figure S10).

### Diet-Induced Obesity and Hyperlipidemia in Mice Promoted by *T. asahii* Y2

**Figure 5:**
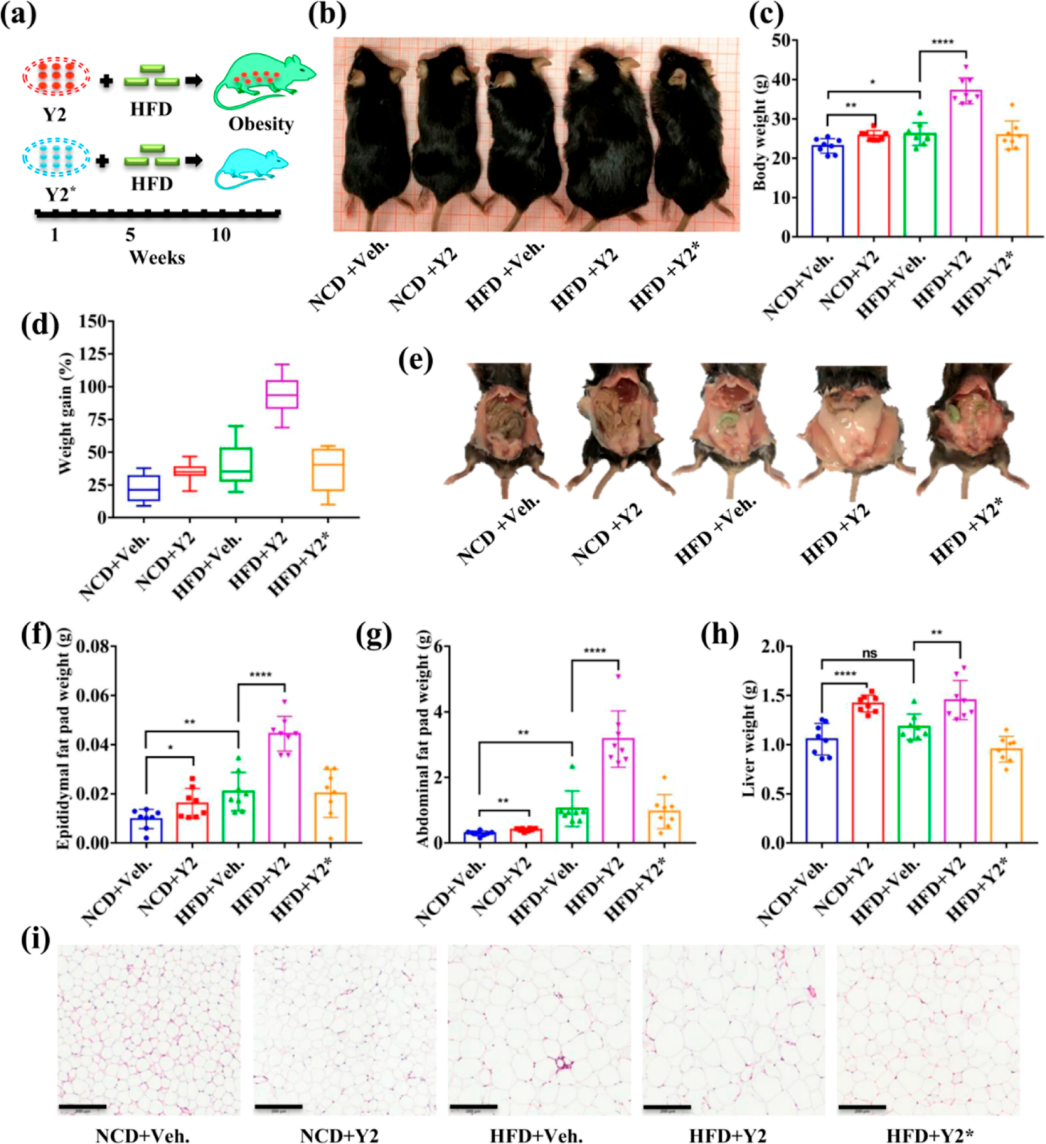
Biological effect of *T. asahii* Y2 in mice. (a) Schematic diagram of *T. asahii* Y2 colonization and the construction of the HFD-induced mouse model of obesity. Mice were administered with saline or *T. asahii* Y2 (108 cfu/mouse). (b) Apparent imaging of mice. (c) Body weight. (d) Weight gain. (e) Abdominal photographs. (f) Abdominal fat pad weight. (g) Epididymal fat pad weight. (h) Liver weight. (i) H&E staining of abdominal fat tissue, scale bar 200 μm. NCD + Veh (mice fed with NCD and administrated with saline), NCD + Y2 (mice fed with NCD and administrated with *T. asahii* Y2, 10^8^ cfu/mouse), HFD + Veh (mice fed with HFD and administrated with saline), HFD + Y2 (mice fed with HFD mice and administrated with *T. asahii* Y2, 10^8^ cfu/mouse), and HFD + Y2* (mice fed with HFD and administrated with pasteurized *T. asahii* Y2, 10^8^ cfu/mouse). *p < 0.05; **p < 0.01; ***p < 0.001. NCD, normal chow diet. HFD, high fat diet. Veh. saline. *N* = 8.

Research has indicated that lipase and bile acids in the digestive tract are particularly important for lipid digestion and absorption and are closely associated with obesity, hyperlipidemia, and diabetes. Therefore, *T. asahii* Y2 as a lipase active intestinal fungus, and its ability to influence lipid metabolism attracted our interest. Therefore, we used mice fed with normal chow or high fat diet treated with *T. asahii* Y2 for 13 weeks to evaluate the development of obesity and hyperlipidemia (Figure 5a). Pasteurized *T. asahii* Y2 (30 min at 121 °C) was administrated to mice as a control experiment. In general, both NCD (normal chow diet) and HFD (high fat diet) fed mice treated with *T. asahii* Y2 have larger body sizes than saline treated mice, and the pasteurized *T. asahii* Y2* treated mice (Figure 5b), which was revealed by the body weight. For NCD fed mice (Figure 5c), *T. asahii* Y2 treatment promoted weight gain (34.79%) in comparison with the control group (weight gain 22.57%). However, for the HFD fed mice, *T. asahii* Y2 treated mice exhibited a 92.44% weight gain (Figure 5d). On the other hand, pasteurized *T. asahii* Y2 treated mice displayed a similar weight gain to that of the saline group. Corresponding to the weight gains, *T. asahii* Y2 treated mice developed more fat in the abdominal and epididymis tissues (Figure 5e–g). In addition, increased liver weight was observed for *T. asahii* Y2 treated mice (Figure 5h). In the abdominal fat tissue, adipocyte hypertrophy was observed for *T. asahii* Y2 treated mice, which exhibited the largest diameter of adipocytes in comparison with those from other groups (Figure 5i). Thus, *T. asahii* Y2 treated mice exhibited significant symptoms of obesity.

Once obesity in mice had been induced, the lipid metabolism was then investigated. Therefore, the blood lipids were determined, which indicated that the *T. asahii* Y2 treated mice possessed the highest levels of total cholesterol (T-CHO), total glyceride (TG), low density lipoprotein (LDL-C), and apolipoprotein B (ApoB) (Figure S12a–d), suggesting hyperlipidemia for *T. asahii* Y2 treated mice. Accordingly, it was deduced that *T. asahii* Y2 may interfere with lipid digestion and absorption as well as promote obesity. Herein, the lipase activity of mice feces was measured. Compared with HFD fed mice, the NCD fed mice exhibited lower lipase activity, and *T. asahii* Y2 increased the lipase activity (Figure S12e). For HFD fed mice, pasteurized *T. asahii* Y2 treated mice exhibit similar lipase activity to the vehicle group, and *T. asahii* Y2 treated mice have the largest lipase activity. Therefore, it was obvious that *T. asahii* Y2 could increase lipase activity in the gut of mice, which may result in disorders of lipid metabolism.

Previous studies revealed that HFD-fed obese mice produced higher levels of pro-inflammatory cytokines in hepatic and adipose tissues, such as tumor necrosis factor-alpha (TNF-α) and interleukin-1β (IL-1β). Therefore, we measured the levels of secreted TNF-α and IL-1β proteins in hepatic and epididymal fat tissues of mice after 13 weeks of HFD feeding with the administration of vehicle, live *T. asahii* Y2, and pasteurized *T. asahii* Y2. All HFD fed mice exhibited higher levels of these cytokines than those of NCD fed mice, induced by obesity. For the obese mice treated by live *T. asahii* Y2, the largest amount of pro-inflammatory cytokines was determined in hepatic and epididymal fat tissues (Figure S12f–i).

The liver is an important organ for the metabolism of lipids. As such pathological analysis of hepatic tissue sections indicated disfunction of lipid metabolism. Herein, the oil red O stained hepatic sections indicated that the most lipids existed in the liver cells of mice treated with *T. asahii* Y2 (Figure S12j). Similarly, H&E stained hepatic sections indicated the tissue lesion of mice administrated with *T. asahii* Y2 (Figure S12k).

From the above results, it is clear that *T. asahii* Y2 an intestinal fungus cultivated from human feces exhibits significant lipase activity. As such, when HFD fed mice were administered with *T. asahii* Y2, the lipase activity in the digestive tract of the mice was enhanced resulting in increased digestion and absorption of lipids, which promoted the disfunction of the lipid metabolism. Consequently, live *T. asahii* Y2 treated mice exhibited significant obesity, hyperlipidemia, inflammation, and non-alcoholic fatty liver disease.

## Conclusions

Lipase from gut microbes and the pancreas influence the digestion and absorption of lipids, and as such lipase activity is correlated with obesity. Therefore, we developed **DDAO-C6** as a NIR fluorescent probe for lipase activity, exhibiting high selectivity and sensitivity. Significantly, **DDAO-C6** could be used as part of a bioassay for the detection of lipase in human feces. In addition, **DDAO-C6** could be used to sense lipase activity and visually identify intestinal fungi, which led to the cultivation of *T. asahii* Y2, a lipase active fungus from human feces. Finally, using HFD induced obese mice, the role of *T. asahii* Y2 in obesity, hyperlipidemia, and inflammation was evaluated. In summary, **DDAO-C6** exhibited great potential in the assay of lipase activity in real time and facilitated the visual identification of intestinal fungi. Therefore, we anticipate that **DDAO-C6** could be used to help diagnosis and treat obesity, hyperlipidemia, and other disorders of lipid metabolism.

## Supporting Information Available

Synthesis scheme and characterization of compounds, spectroscopy, fluorescence behavior of **DDAO-C6**, and bioimaging data (PDF)
