# Supplementary material for: Visual Identification of Trichosporon asahii, a Gut Yeast Associated with Obesity, Using an Enzymatic NIR Fluorescent Probe
Source: Anal Chem. 2022 Aug 3;94(32):11216–23. doi: 10.1021/acs.analchem.2c01691 (PMC9386680; doi:10.1021/acs.analchem.2c01691)
Supplement: Supplementary file 1 — ac2c01691_si_001.pdf [file ac2c01691_si_001.pdf]

Supporting information

Visual Identification of Trichosporon asahii, a Gut Yeast Associated with Obesity, Using an Enzymatic NIR Fluorescent Probe

Lei Feng,[†,‡,§,#] Ying Deng,[‡,#] Shufan Song,[‡] Yanqiu Sun,[‡] Jingnan Cui,[※] Xiaochi Ma,[†,‡,*] Lingling Jin,[‡] Yan Wang,[‡] Tony D. James,[§,¤,*] Chao Wang[†,‡,*]

[†] *Second Affiliated Hospital, Dalian Medical University, Dalian 116023, China*

[‡] *Dalian Key Laboratory of Metabolic Target Characterization and Traditional Chinese Medicine Intervention, College of Pharmacy, College of Integrative Medicine, Dalian Medical University, Dalian 116044, China*

[§]*School of Chemistry and Chemical Engineering, Henan Normal University, Xinxiang 453007, China*

[※]*State Key Laboratory of Fine Chemicals, Dalian University of Technology, Dalian 116024, China*

[¤] *Department of Chemistry, University of Bath, Bath BA2 7AY, UK*

* *Corresponding authors: maxc1978@163.com (X. Ma), t.d.james@bath.ac.uk (T.D. James), wach_edu@sina.com (C. Wang)*

[#] *These authors contributed equally to this work.*

Index

Synthesis of fluorescent probes 4
Scheme S1. Synthetic route for **DDAO-C6**, **-C8**, **-C10**, **-C12**. 4
Scheme S2. Synthetic route for **DDAO-C14**, **-C15**, **-C16**, **-C18**, **-C20**. 5
Figure S1. HPLC chromatograms for the hydrolysis of **DDAO-C6** mediated by lipase.. 7
Figure S2. Linear relationship between the fluorescence intensity at 658 nm and lipase activity for the enzymatic hydrolysis of **DDAO-C6** by lipase. 7
Figure S3. (a) Fluorescence behavior for the hydrolysis of **DDAO-C6** by lipase with different incubation time. (b) Linear relationship between the fluorescence intensity at 658 nm and incubation time for the enzymatic hydrolysis of **DDAO-C6** by lipase........ 8
Figure S4. The fluorescence responses of **DDAO** and **DDAO-C6** in physiological saline with different pH values (2-12)........ 8
Figure S5. Fluorescence responses of **DDAO-C6** toward various active proteins. 9
Figure S6. Fluorescence responses of **DDAO-C6** toward various species (amino acids, ions, and ROS). 9
Figure S7. HPLC analysis for the metabolism of **DDAO-C6** by *Trichosporon asahii* Y2. (a) Reference standard **DDAO-C6**. (b) Metabolite analysis of **DDAO-C6** in *Trichosporon asahii* Y2. (c) Reference standard **DDAO**. (d) Analysis about the fermentation of *Trichosporon asahii* Y2. Detection wavelength 450 nm........ 10
Figure S8. Fluorescence responses of **DDAO-C6** toward *Trichosporon asahii* Y2 at different concentrations. 10
Figure S9. Fluorescence response of **DDAO-C6** towards *Trichosporon asahii* Y2 co-incubated with orlistat. 11
Figure S10. Fluorescence response of **DDAO-C6** toward *Trichosporon asahii* Y2 at different growth stages. 11
Figure S11. (a) Blood glucose curve and (b) area under the curve in OGTT. 12
Figure S12. Evaluation of lipid metabolism for the mice.. 13
Figure S13. ^{1}H NMR spectrum of **DDAO-C6**. 14
Figure S14. ^{13}C NMR spectrum of **DDAO-C6**. 14
Figure S15. HR-ESI-MS of **DDAO-C6**. 15
Figure S16. ^{1}H NMR spectrum of **DDAO-C8**. 15
Figure S17. ^{13}C NMR spectrum of **DDAO-C8**. 16
Figure S18. HR-ESI-MS of **DDAO-C8**. 16
Figure S19. ^{1}H NMR spectrum of **DDAO-C10**. 17
Figure S20. ^{13}C NMR spectrum of **DDAO-C10**. 17
Figure S21. HR-ESI-MS of **DDAO-C10**. 18
Figure S22. ^{1}H NMR spectrum of **DDAO-C12**. 18
Figure S23. ^{13}C NMR spectrum of **DDAO-C12**. 19
Figure S24. HR-ESI-MS of **DDAO-C12**. 19
Figure S25. ^{1}H NMR spectrum of **DDAO-C14**. 20
Figure S26. ^{13}C NMR spectrum of **DDAO-C14**. 20
Figure S27. HR-ESI-MS spectrum of **DDAO-C14**. 21
Figure S28. ^{1}H NMR spectrum of **DDAO-C15**. 21
Figure S29. ^{13}C NMR spectrum of **DDAO-C15**. 22

Figure S30. HR-ESI-MS of **DDAO-C15**. 22
Figure S31. ^{1}H NMR spectrum of **DDAO-C16**. 23
Figure S32. ^{13}C NMR spectrum of **DDAO-C16**. 23
Figure S33. HR-ESI-MS spectrum of **DDAO-C16**. 24
Figure S34. ^{1}H NMR spectrum of **DDAO-C18**. 24
Figure S35. ^{13}C NMR spectrum of **DDAO-C18**. 25
Figure S36. HR-ESI-MS spectrum of **DDAO-C18**. 25
Figure S37. ^{1}H NMR spectrum of **DDAO-C20**. 26
Figure S38. ^{13}C NMR spectrum of **DDAO-C20**. 26
Figure S39. HR-ESI-MS spectrum of **DDAO-C20**. 27

Synthesis of fluorescent probes

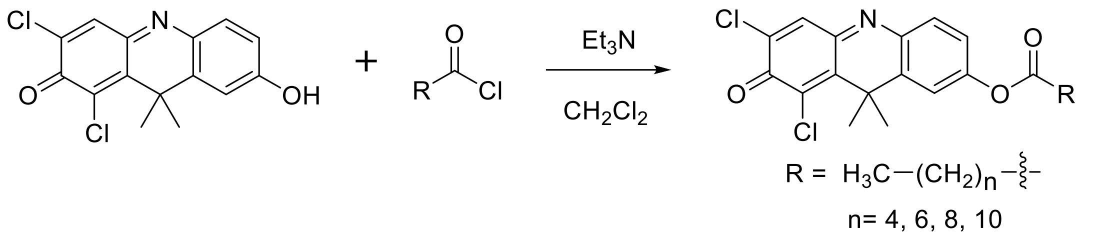

Scheme S1. Synthetic route for **DDAO-C6**, **-C8**, **-C10**, **-C12**.

To a solution of 0.1 mmol **DDAO** and 0.15 mmol Et_3N in 10 mL of CH_2Cl_2, acyl chloride (0.12 mmol, mixed with 1 mL of CH_2Cl_2) was added dropwise at 0 °C in 30 min. After stirring at this temperature for 1 h, the mixture was warmed to room temperature and stirred overnight. The solvent was removed *in vacuo*, and the residual solid was purified by chromatography (silica gel, EtOAc–hexane as eluent, 1: 5, *V*/*V*) to afford an orange solid. The structure was confirmed by ^{1}H NMR, ^{13}C NMR and HRMS.

DDAO-C6 (n=4, 15.4 mg, yield 38%). ^{1}H NMR (600 MHz, $CDCl_3$) δ 7.67 (1H, d, J = 8.4 Hz), 7.64 (1H, s), 7.23 (1H, d, J = 2.4 Hz), 7.13 (1H, dd, J = 8.4, 2.4 Hz), 2.60 (1H, t, J = 7.2 Hz), 1.89 (6H, s), 1.78 (2H, m), 1.41 (4H, m), 0.95 (3H, t, J = 7.2 Hz). ^{13}C NMR (150 MHz, $CDCl_3$) δ 173.19, 171.75, 153.46, 149.74, 140.35, 139.44, 139.43, 138.49, 137.35, 135.54, 133.07, 121.49, 119.81, 39.08, 34.38, 31.24, 26.65, 24.47, 22.31, 13.91. HR-ESI-MS *m/z* 406.0796 $[M+H]^+$, calcd for $C_{21}H_{22}Cl_2NO_3^+$ 406.0791.

DDAO-C8 (n=6, 17.8 mg, yield 41%). ^{1}H NMR (600 MHz, $CDCl_3$) δ 7.67 (1H, d, J = 8.4 Hz), 7.64 (1H, s), 7.23 (1H, d, J = 2.4 Hz), 7.13 (1H, dd, J = 8.4, 2.4 Hz), 2.60 (1H, t, J = 7.8 Hz), 1.88 (6H, s), 1.77 (2H, m), 1.43 (8H, m), 0.90 (3H, t, J = 6.6 Hz). ^{13}C NMR (150 MHz, $CDCl_3$) δ 173.20, 171.77, 153.46, 149.74, 140.35, 139.45, 139.43, 138.49, 135.54, 133.08, 121.51, 119.82, 39.08, 34.42, 31.64, 29.05, 28.91, 26.64, 24.79, 22.61, 14.08. HR-ESI-MS *m/z* 434.1280 $[M+H]^+$, calcd for $C_{23}H_{26}Cl_2NO_3^+$ 434.1284.

DDAO-C10 (n=8, 17.3 mg, yield 37.4%). ^{1}H NMR (600 MHz, $CDCl_3$) δ 7.67 (1H, d, J = 8.4 Hz), 7.64 (1H, s), 7.23 (1H, d, J = 2.4 Hz), 7.13 (1H, dd, J = 8.4, 2.4 Hz), 2.60 (1H, t, J = 7.8 Hz), 1.88 (6H, s), 1.77 (2H, m), 1.25-1.45 (12H, m), 0.89 (3H, t, J = 7.2 Hz). ^{13}C NMR (150 MHz, $CDCl_3$) δ 173.21, 171.78, 153.46, 149.75, 140.35,

139.46, 139.43, 138.50, 137.36, 135.54, 133.09, 121.52, 119.83, 39.08, 34.43, 31.87, 29.42, 29.26, 29.10, 26.65, 24.80, 22.68, 14.13. HR-ESI-MS *m/z* 462.1600 $[M+H]^+$, calcd for $C_{25}H_{30}Cl_2NO_3^+$ 462.1597.

DDAO-C12 (n=10, 20.2 mg, yield 41.2%). ^{1}H NMR (600 MHz, $CDCl_3$) δ 7.67 (1H, d, J = 8.4 Hz), 7.64 (1H, s), 7.23 (1H, d, J = 2.4 Hz), 7.13 (1H, dd, J = 8.4, 2.4 Hz), 2.60 (1H, t, J = 7.8 Hz), 1.88 (6H, s), 1.77 (2H, m), 1.25-1.43 (14H, m), 0.88 (3H, t, J = 7.2 Hz). ^{13}C NMR (150 MHz, $CDCl_3$) δ 173.21, 171.78, 153.46, 149.75, 140.35, 139.46, 139.43, 138.49, 137.36, 135.54, 133.09, 121.52, 119.83, 39.08, 34.43, 31.91, 29.61, 29.46, 29.35, 29.26, 29.10, 26.65, 24.80, 22.70, 14.13. HR-ESI-MS *m/z* 490.1909 $[M+H]^+$, calcd for $C_{27}H_{34}Cl_2NO_3^+$ 490.1910.

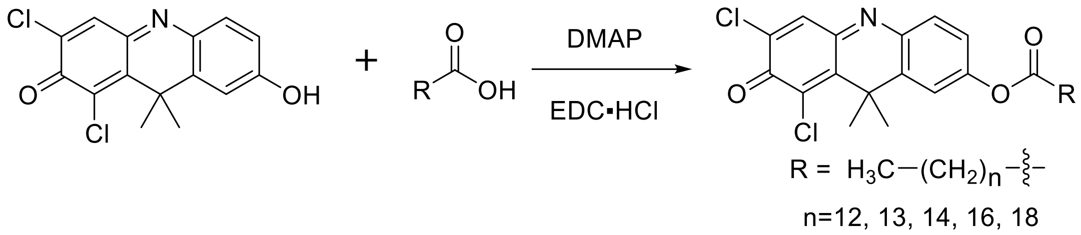

Scheme S2. Synthetic route for **DDAO-C14**, **-C15**, **-C16**, **-C18**, **-C20**.

To a solution of 0.1 mmol **DDAO** and 1.5 mmol fatty acid in 20 mL of CH_2Cl_2, 0.1 mmol DMAP and 0.5 mmol EDC·HCl were added, then the mixture was stirred overnight. The solvent was removed *in vacuo*, and the residual solid was purified by chromatography (silica gel, EtOAc–hexane as eluent, 1: 5, *V*/*V*) to afford an orange solid. The structure was confirmed by ^{1}H NMR, ^{13}C NMR and HRMS.

DDAO-C14 (n=12, 15.4 mg, yield 29.7%). ^{1}H NMR (600 MHz, $CDCl_3$) δ 7.67 (1H, d, J = 8.4 Hz), 7.64 (1H, s), 7.23 (1H, d, J = 2.4 Hz), 7.13 (1H, dd, J = 8.4, 2.4 Hz), 2.60 (1H, t, J = 7.8 Hz), 1.88 (6H, s), 1.77 (2H, m), 1.25-1.44 (20H, m), 0.88 (3H, t, J = 7.2 Hz). ^{13}C NMR (150 MHz, $CDCl_3$) δ 173.22, 171.79, 153.46, 149.75, 140.36, 139.46, 139.43, 138.50, 137.36, 135.55, 133.09, 121.52, 119.83, 39.08, 34.43, 31.93, 29.69, 29.66, 29.61, 29.46, 29.37, 29.25, 29.10, 26.65, 24.80, 22.70, 14.14. HR-ESI-MS *m/z* 518.2219 $[M+H]^+$, calcd for $C_{29}H_{38}Cl_2NO_3^+$ 518.2223.

DDAO-C15 (n=13, 13.9 mg, yield 26.1%). ^{1}H NMR (600 MHz, $CDCl_3$) δ 7.67 (1H, d, J = 8.4 Hz), 7.64 (1H, s), 7.23 (1H, d, J = 2.4 Hz), 7.13 (1H, dd, J = 8.4, 2.4 Hz), 2.60 (1H, t, J = 7.8 Hz), 1.88 (6H, s), 1.77 (2H, m), 1.26-1.43 (22H, m), 0.88 (3H, t, J = 7.2 Hz). ^{13}C NMR (150 MHz, $CDCl_3$) δ 173.21, 171.78, 153.46, 149.75, 140.35, 139.46, 139.43, 138.50, 137.36, 135.55, 133.09, 121.52, 119.83, 39.08, 34.43, 31.93, 29.69, 29.66, 29.61, 29.46, 29.37, 29.25, 29.10, 26.65, 24.80, 22.70, 14.14. HR-ESI-MS *m/z* 532.2376 $[M+H]^+$, calcd for $C_{30}H_{40}Cl_2NO_3^+$ 532.2380.

DDAO-C16 (n=14, 13.5 mg, yield 24.7%). ^{1}H NMR (600 MHz, $CDCl_3$) δ 7.67 (1H, d, J = 8.4 Hz), 7.64 (1H, s), 7.23 (1H, d, J = 2.4 Hz), 7.13 (1H, dd, J = 8.4, 2.4 Hz), 2.60 (1H, t, J = 7.8 Hz), 1.88 (6H, s), 1.77 (2H, m), 1.26-1.44 (24H, m), 0.88 (3H, t, J = 7.2 Hz). ^{13}C NMR (150 MHz, $CDCl_3$) δ 173.20, 171.77, 153.48, 149.75, 140.37, 139.46, 139.44, 138.50, 137.37, 135.55, 133.09, 121.51, 119.82, 39.09, 34.44, 31.93, 29.70, 29.66, 29.61, 29.46, 29.37, 29.26, 29.11, 26.67, 24.81, 22.70, 14.13. HR-ESI-MS *m/z* 546.2530 $[M+H]^+$, calcd for $C_{31}H_{42}Cl_2NO_3^+$ 546.2536.

DDAO-C18 (n=16, 16.2 mg, yield 28.2%). ^{1}H NMR (600 MHz, $CDCl_3$) δ 7.67 (1H, d, J = 8.4 Hz), 7.64 (1H, s), 7.23 (1H, d, J = 2.4 Hz), 7.13 (1H, dd, J = 8.4, 2.4 Hz), 2.60 (1H, t, J = 7.8 Hz), 1.88 (6H, s), 1.77 (2H, m), 1.25-1.44 (28H, m), 0.88 (3H, t, J = 7.2 Hz). ^{13}C NMR (150 MHz, $CDCl_3$) δ 173.21, 171.78, 153.46, 149.75, 140.35, 139.46, 139.43, 138.50, 137.36, 135.55, 133.09, 121.52, 119.83, 39.08, 34.43, 31.93, 29.70, 29.66, 29.61, 29.46, 29.37, 29.26, 29.10, 26.65, 24.80, 22.70, 14.14. HR-ESI-MS *m/z* 574.2867 $[M+H]^+$, calcd for $C_{33}H_{46}Cl_2NO_3^+$ 574.2849.

DDAO-C20 (n=18, 20.2 mg, yield 33.6%). ^{1}H NMR (600 MHz, $CDCl_3$) δ 7.67 (1H, d, J = 8.4 Hz), 7.64 (1H, s), 7.23 (1H, d, J = 2.4 Hz), 7.13 (1H, dd, J = 8.4, 2.4 Hz), 2.60 (1H, t, J = 7.8 Hz), 1.88 (6H, s), 1.77 (2H, m), 1.25-1.44 (32H, m), 0.88 (3H, t, J = 7.2 Hz). ^{13}C NMR (150 MHz, $CDCl_3$) δ 173.21, 171.78, 153.46, 149.75, 140.35, 139.46, 139.42, 138.49, 137.36, 135.54, 133.08, 121.51, 119.83, 39.08, 34.43, 31.93, 29.71, 29.67, 29.61, 29.46, 29.37, 29.26, 29.10, 26.65, 24.80, 22.70, 14.14. HR-ESI-MS *m/z* 602.3166 $[M+H]^+$, calcd for $C_{35}H_{50}Cl_2NO_3^+$ 602.3162.

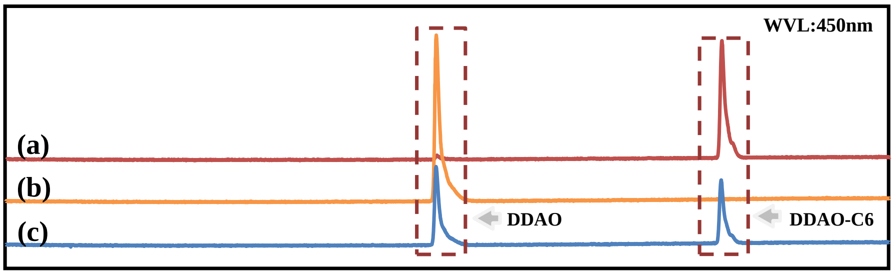

Figure S1. HPLC chromatograms for the hydrolysis of **DDAO-C6** mediated by lipase. (a) Reference standard **DDAO-C6**. (b) Reference standard **DDAO**. (c) Enzymatic hydrolysis of **DDAO-C6** by lipase.

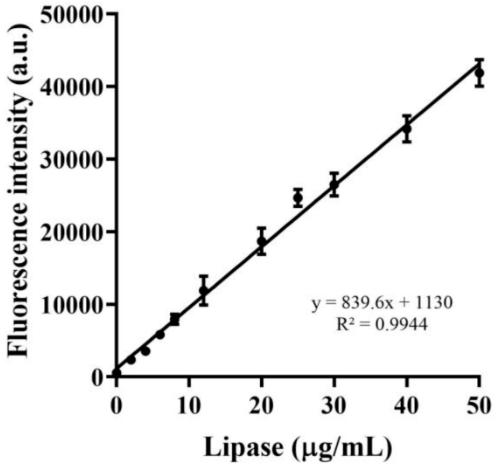

Figure S2. Linear relationship between the fluorescence intensity at 658 nm and lipase activity for the enzymatic hydrolysis of **DDAO-C6** by lipase.

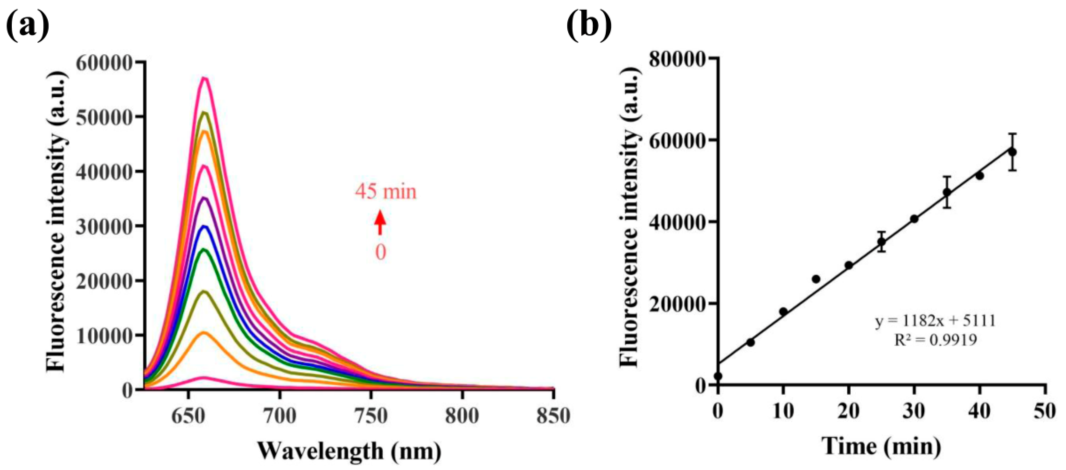

Figure S3. (a) Fluorescence behavior for the hydrolysis of **DDAO-C6** by lipase with different incubation time. (b) Linear relationship between the fluorescence intensity at 658 nm and incubation time for the enzymatic hydrolysis of **DDAO-C6** by lipase.

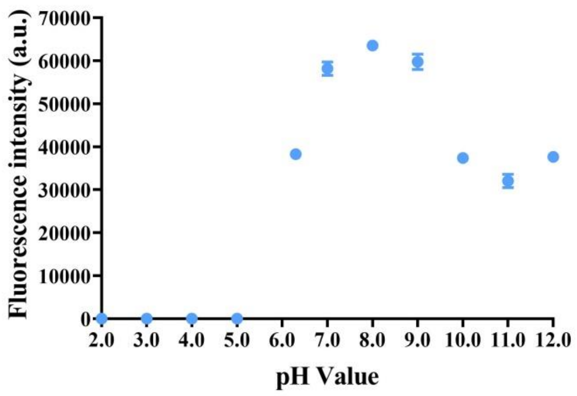

Figure S4. The fluorescence responses of **DDAO** and **DDAO-C6** in physiological saline with different pH values (2-12)

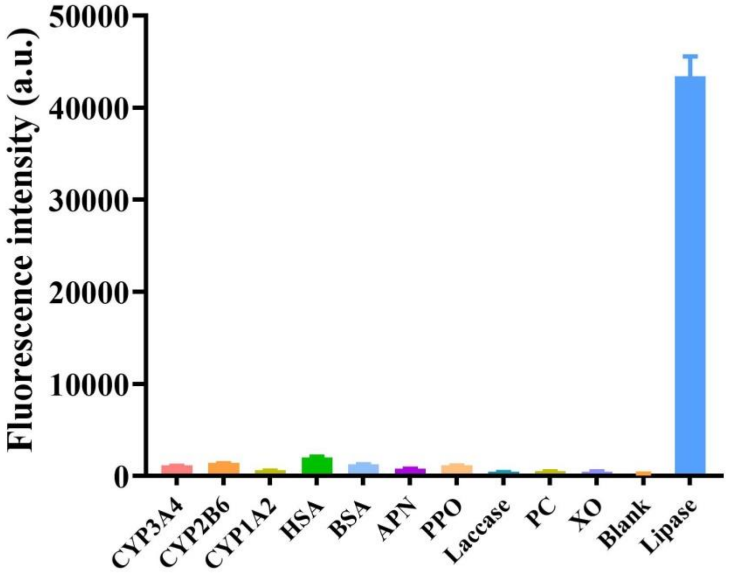

Figure S5. Fluorescence responses of **DDAO-C6** toward various active proteins.

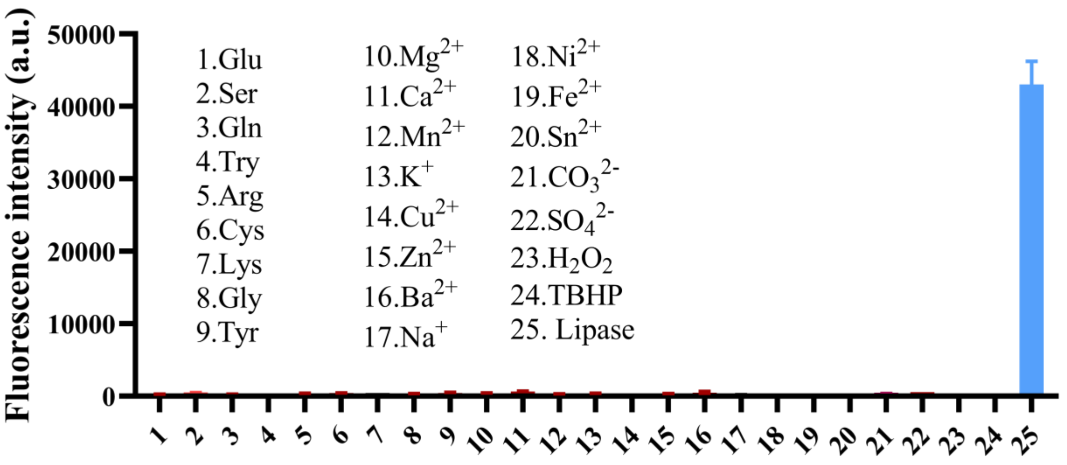

Figure S6. Fluorescence responses of **DDAO-C6** toward various species (amino acids, ions, and ROS).

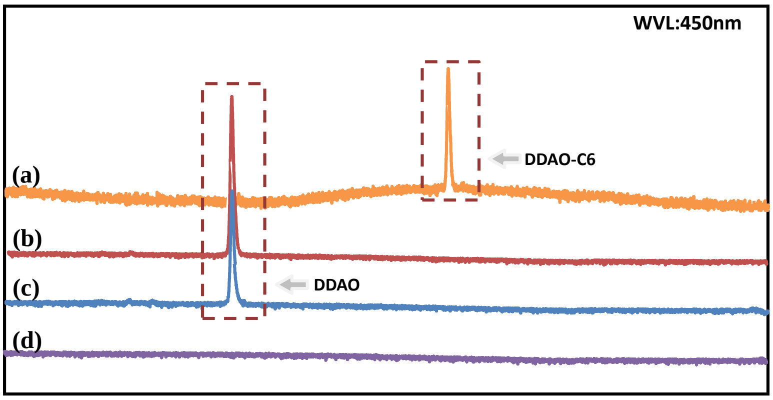

Figure S7. HPLC analysis for the metabolism of **DDAO-C6** by *Trichosporon asahii* Y2. (a) Reference standard **DDAO-C6**. (b) Metabolite analysis of **DDAO-C6** in *Trichosporon asahii* Y2. (c) Reference standard **DDAO**. (d) Analysis about the fermentation of *Trichosporon asahii* Y2. Detection wavelength 450 nm.

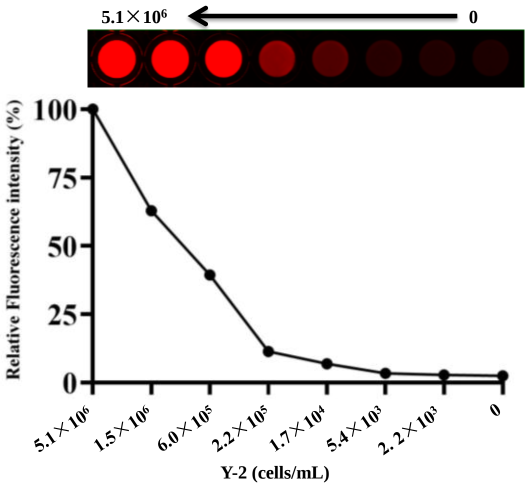

Figure S8. Fluorescence responses of **DDAO-C6** toward *Trichosporon asahii* Y2 at different concentrations.

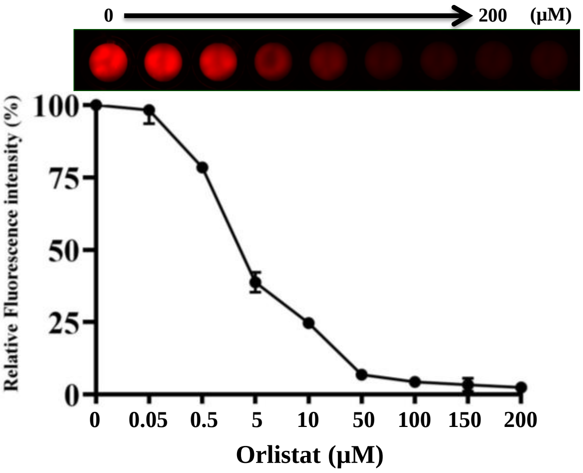

Figure S9. Fluorescence response of **DDAO-C6** towards *Trichosporon asahii* Y2 co-incubated with orlistat.

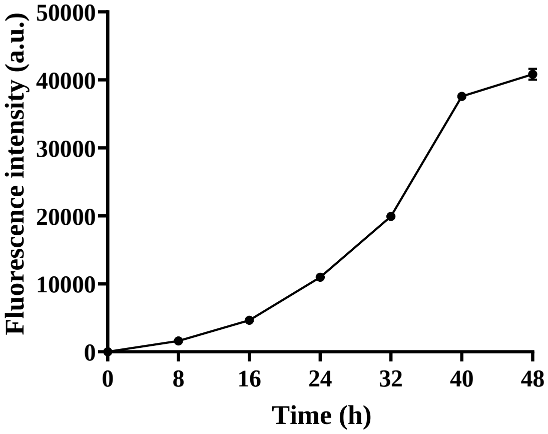

Figure S10. Fluorescence response of **DDAO-C6** toward *Trichosporon asahii* Y2 at different growth stages.

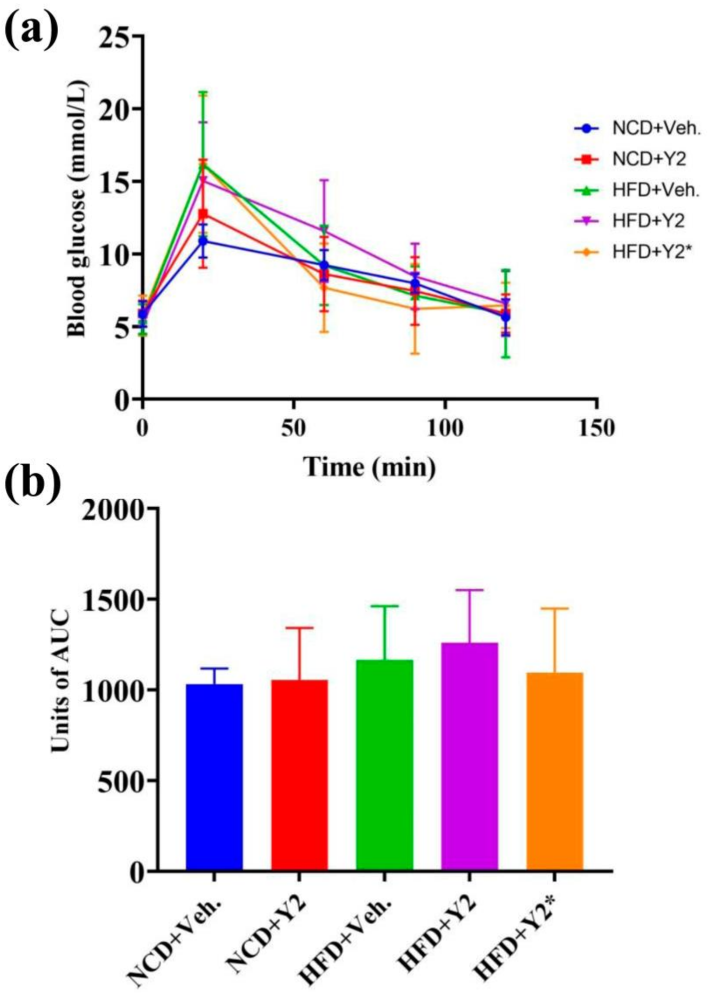

Figure S11. (a) Blood glucose curve and (b) area under the curve in OGTT.

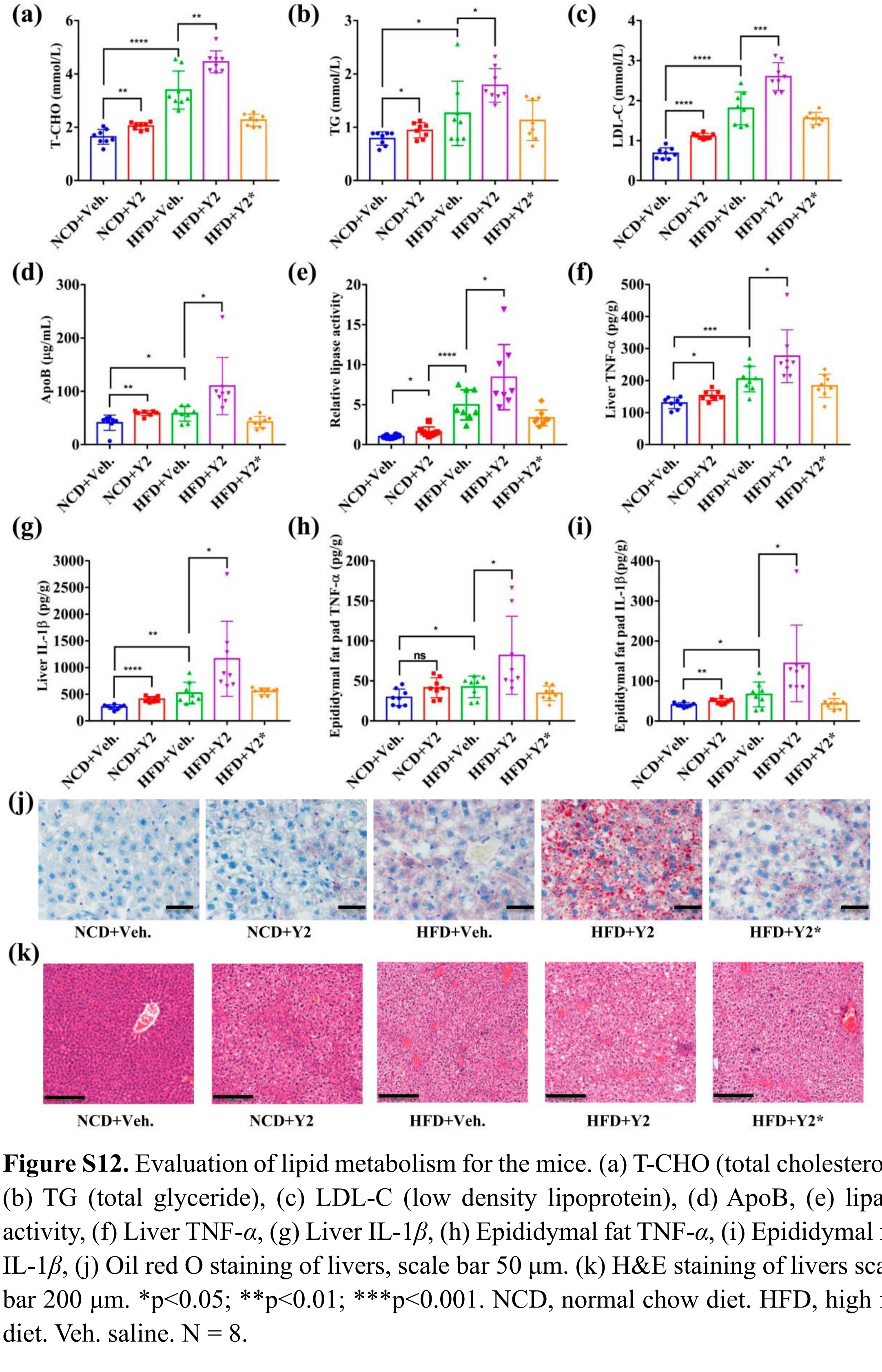

Figure S12. Evaluation of lipid metabolism for the mice. (a) T-CHO (total cholesterol), (b) TG (total glyceride), (c) LDL-C (low density lipoprotein), (d) ApoB, (e) lipase activity, (f) Liver TNF-α, (g) Liver IL-1β, (h) Epididymal fat TNF-α, (i) Epididymal fat IL-1β, (j) Oil red O staining of livers, scale bar 50 μm. (k) H&E staining of livers scale bar 200 μm. *p<0.05; **p<0.01; ***p<0.001. NCD, normal chow diet. HFD, high fat diet. Veh. saline. N = 8.

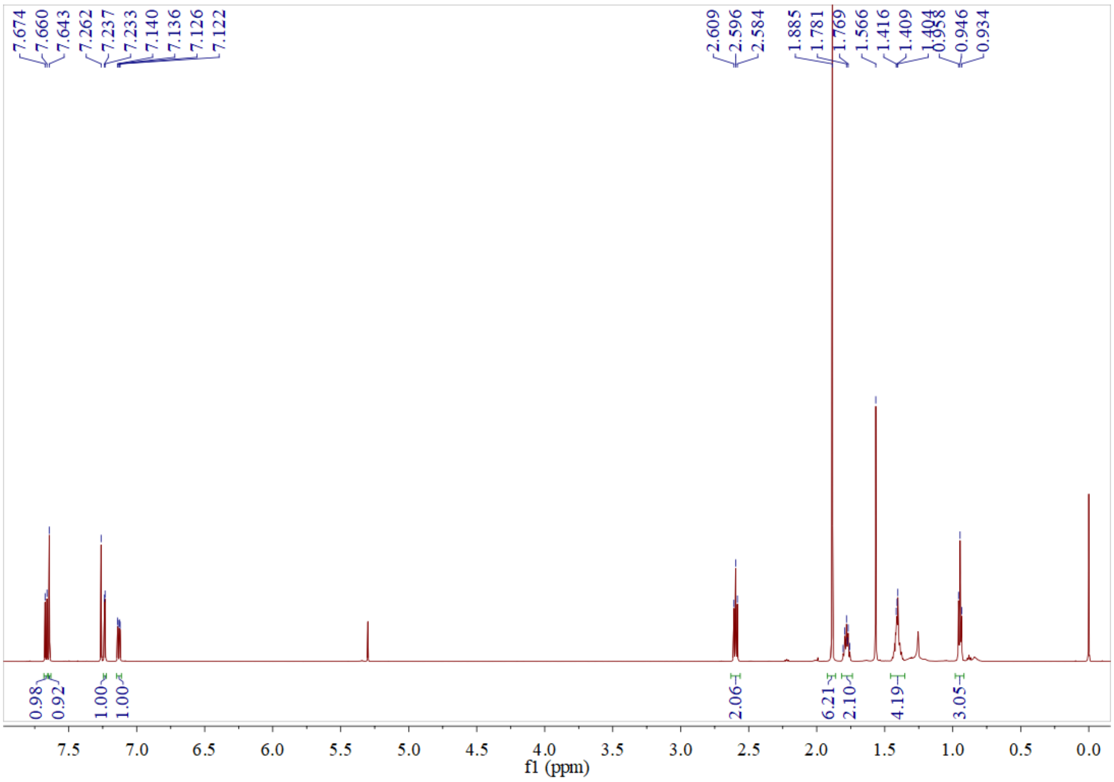

Figure S13. ^{1}H NMR spectrum of **DDAO-C6**.

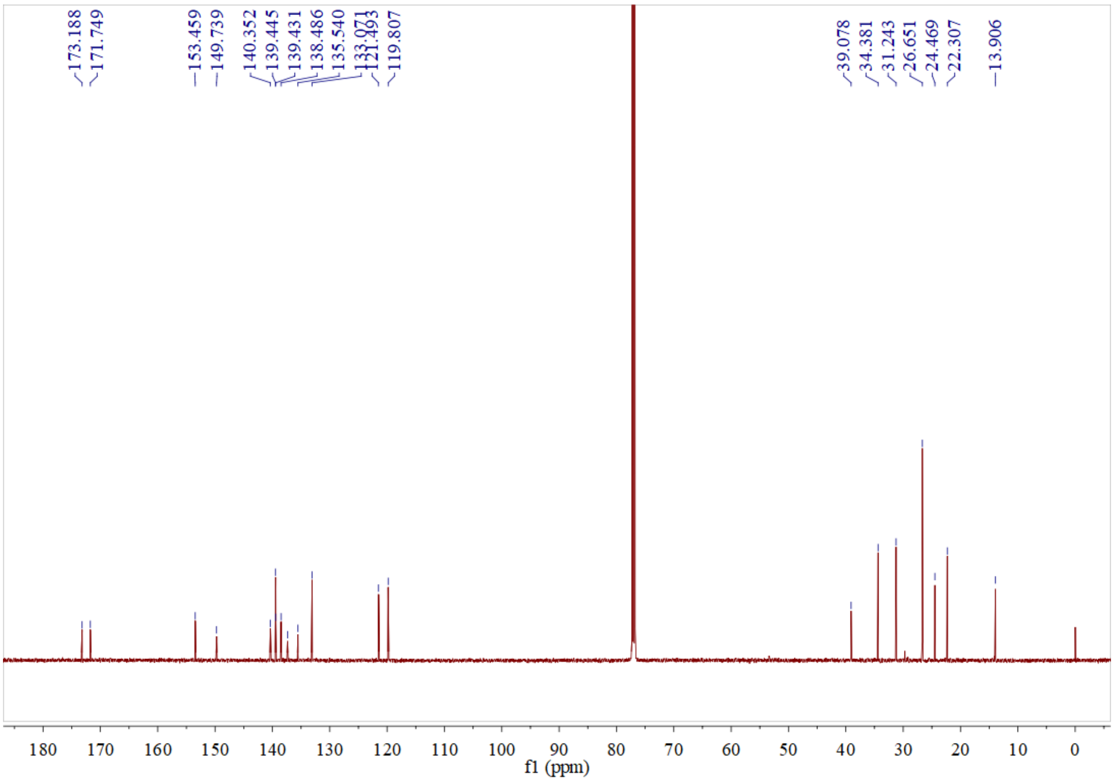

Figure S14. ^{13}C NMR spectrum of **DDAO-C6**.

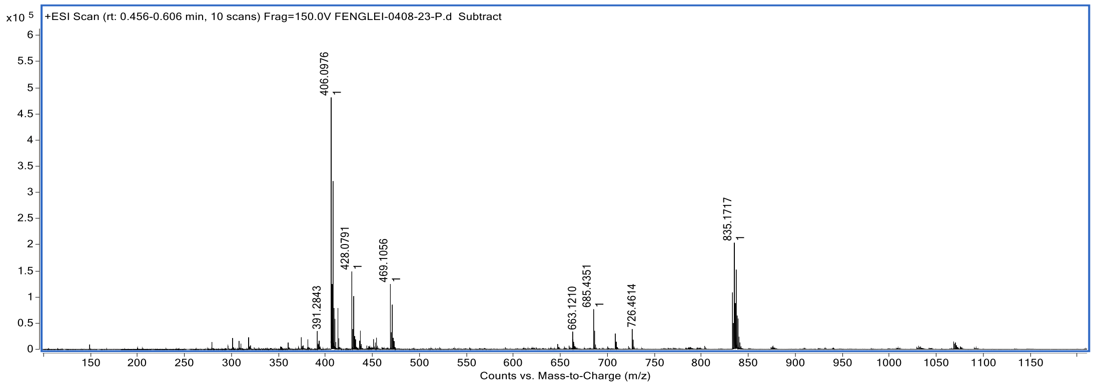

Figure S15. HR-ESI-MS of **DDAO-C6**.

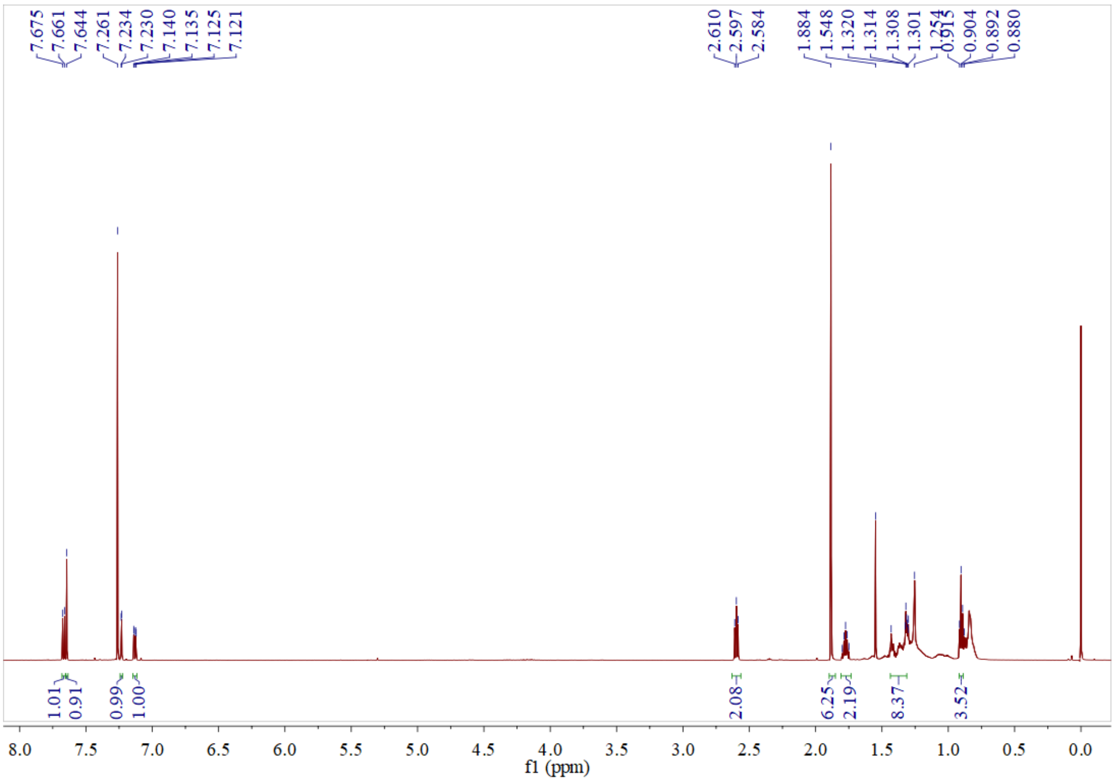

Figure S16. ^{1}H NMR spectrum of **DDAO-C8**.

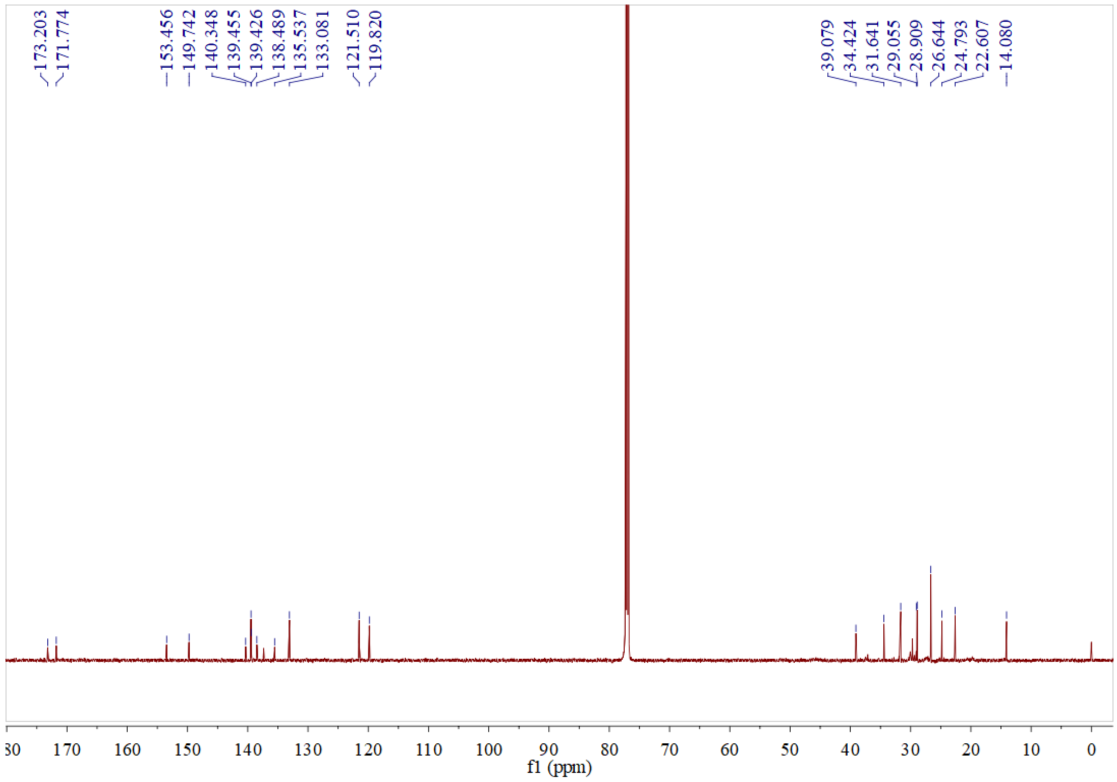

Figure S17. ^{13}C NMR spectrum of **DDAO-C8**.

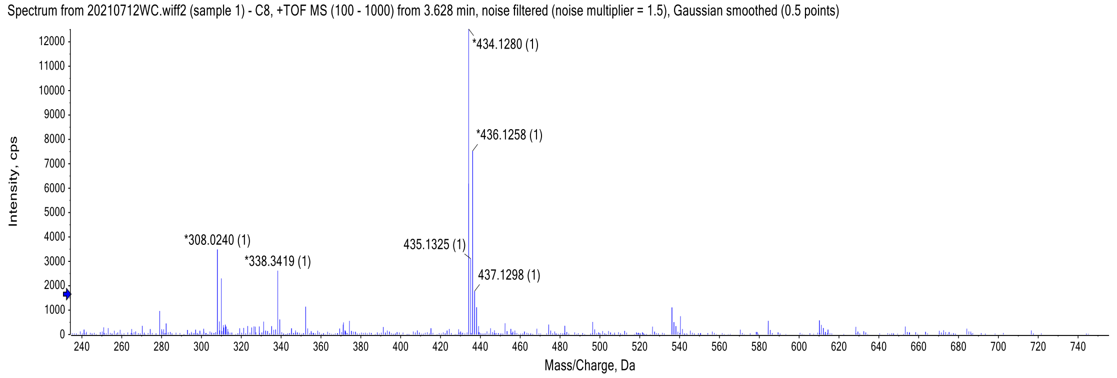

Figure S18. HR-ESI-MS of **DDAO-C8**.

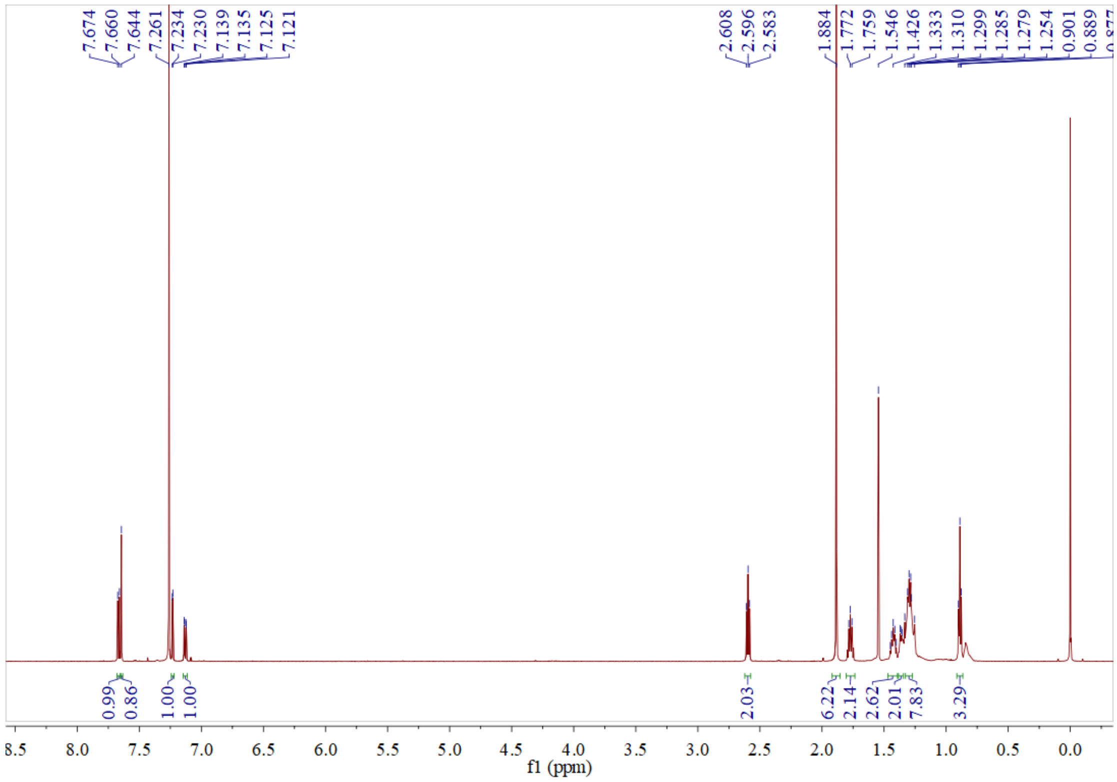

Figure S19. ^{1}H NMR spectrum of **DDAO-C10**.

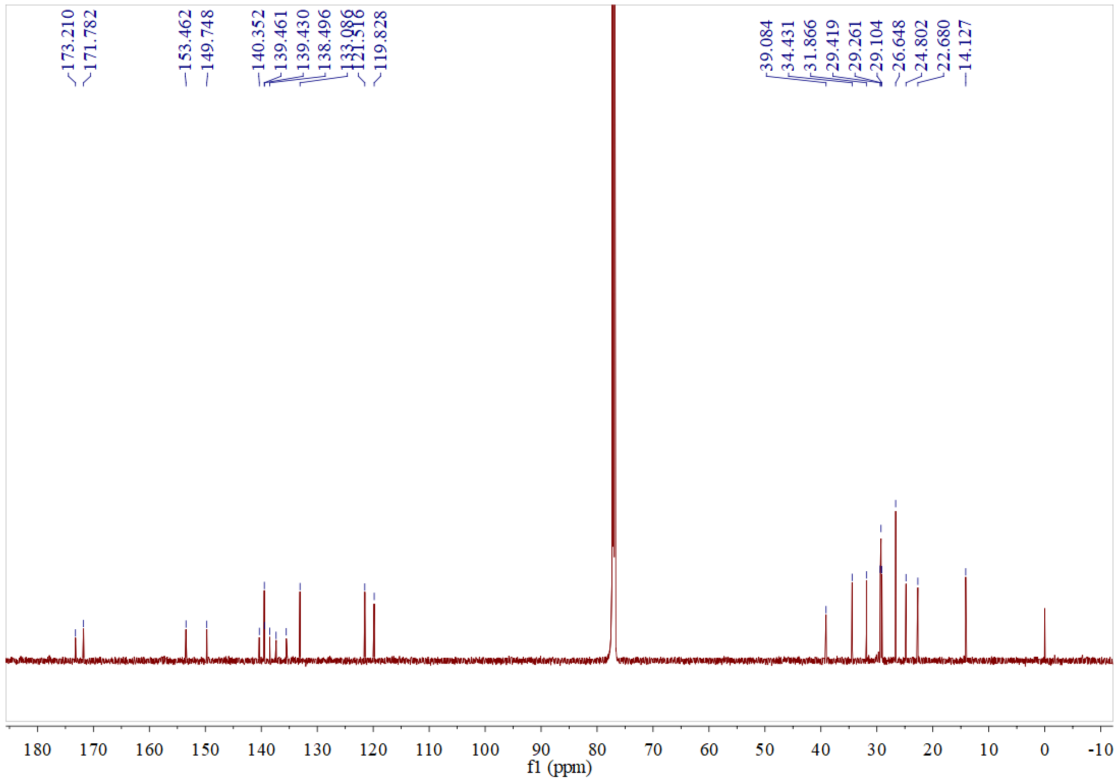

Figure S20. ^{13}C NMR spectrum of **DDAO-C10**.

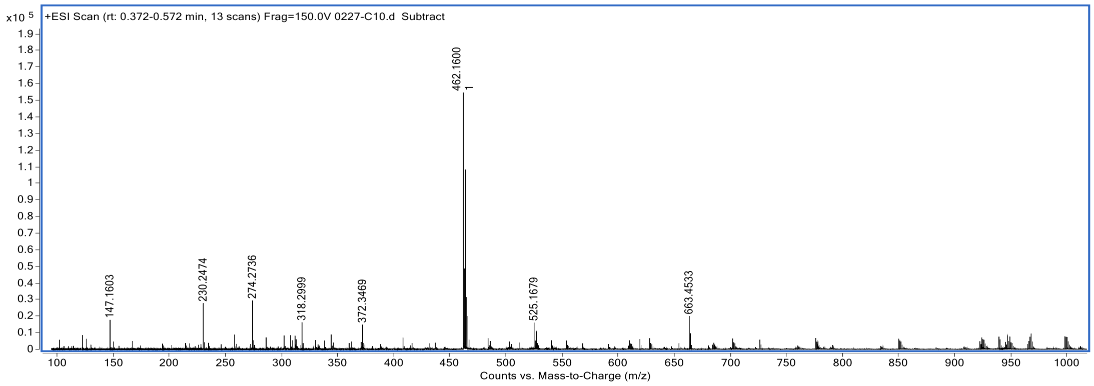

Figure S21. HR-ESI-MS of **DDAO-C10**.

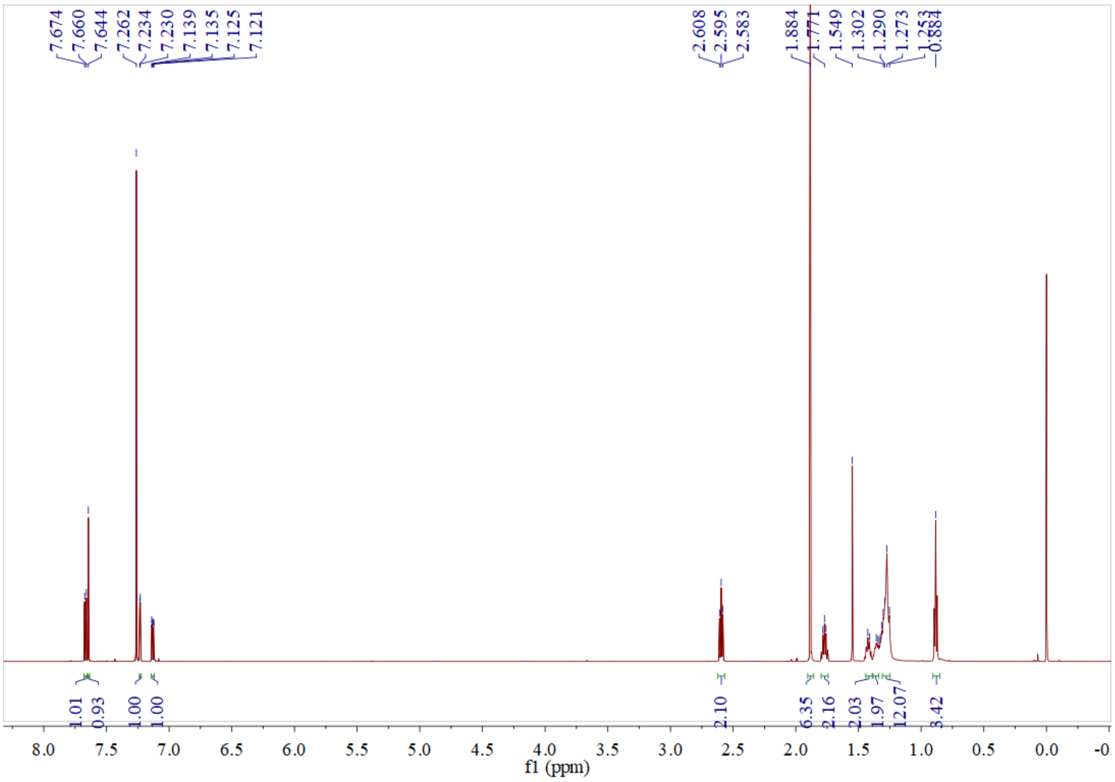

Figure S22. ^{1}H NMR spectrum of **DDAO-C12**.

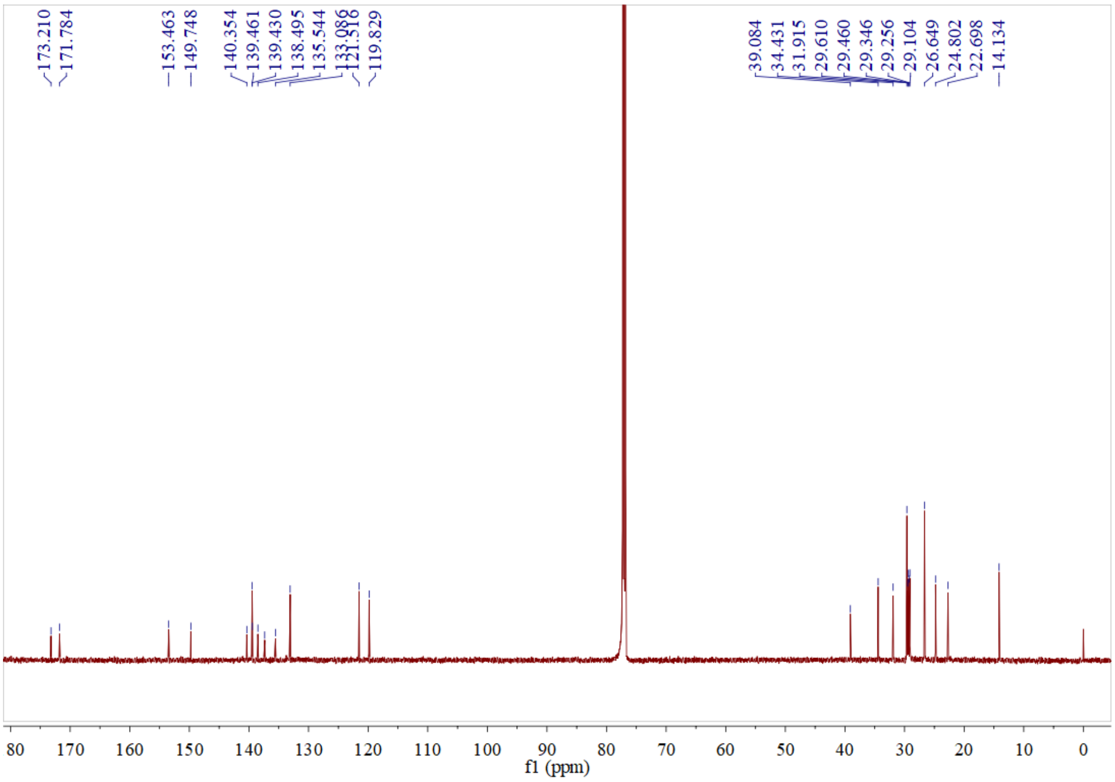

Figure S23. ^{13}C NMR spectrum of **DDAO-C12**.

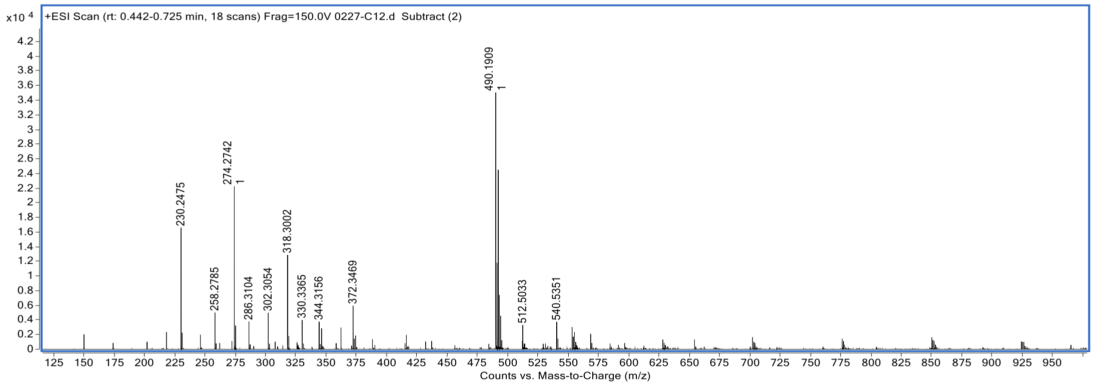

Figure S24. HR-ESI-MS of **DDAO-C12**.

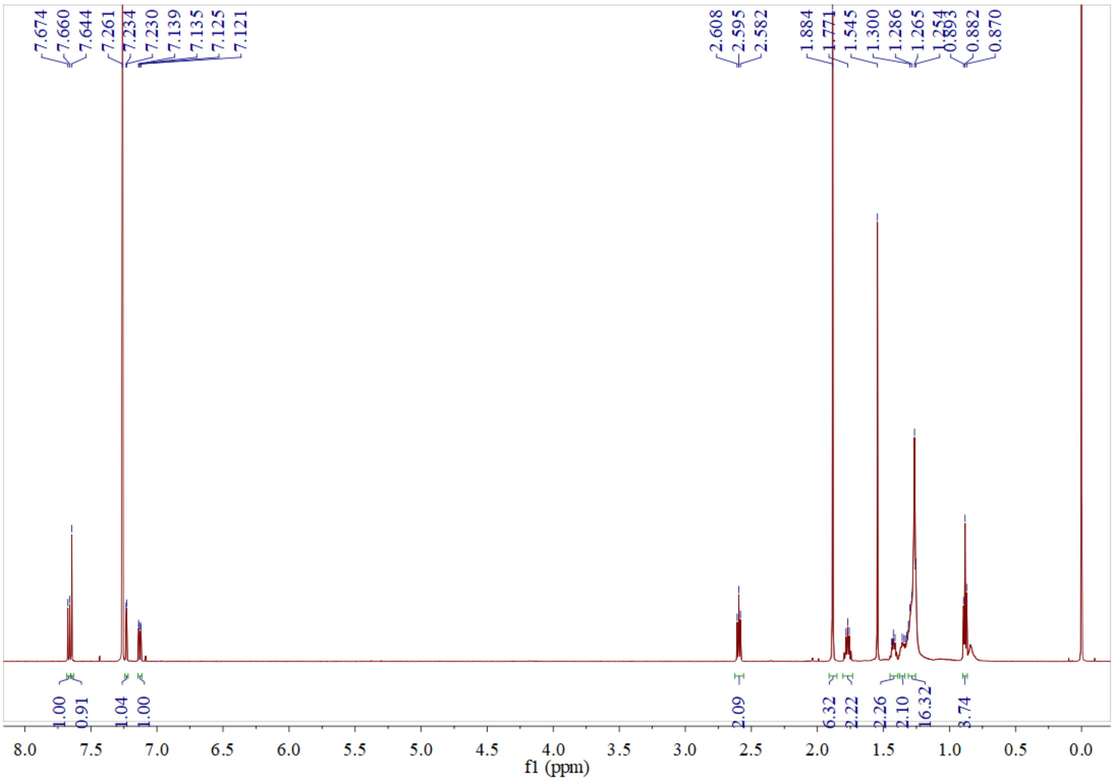

Figure S25. ^{1}H NMR spectrum of **DDAO-C14**.

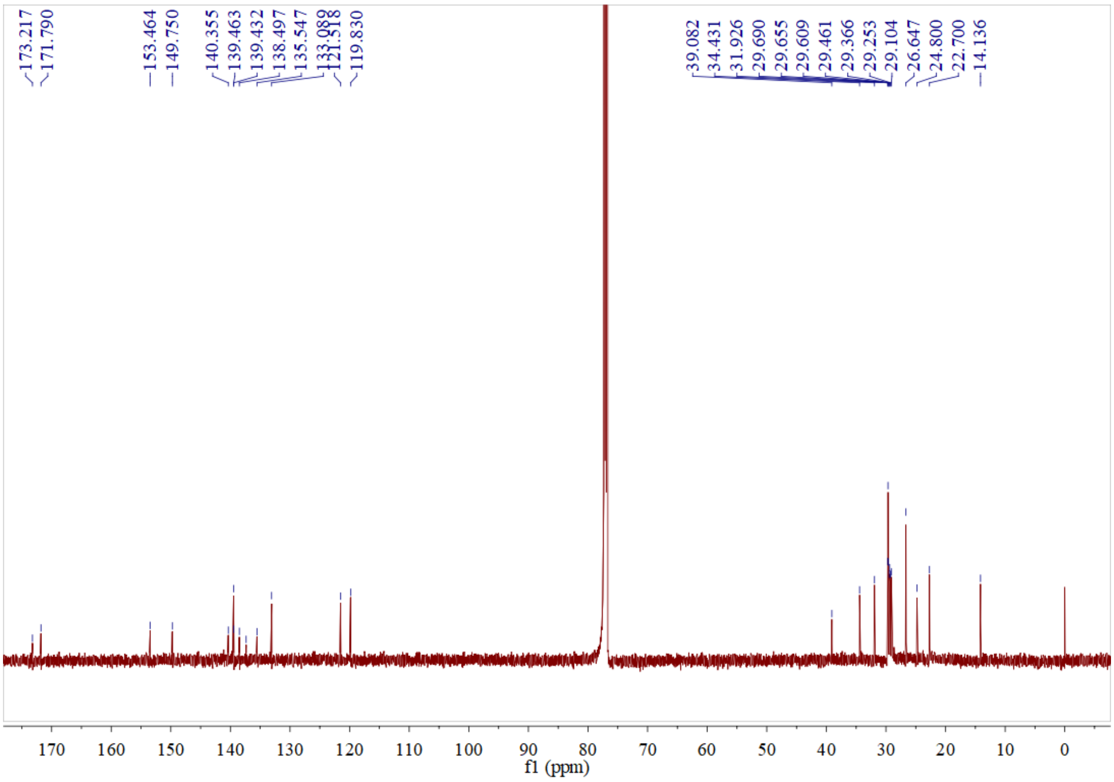

Figure S26. ^{13}C NMR spectrum of **DDAO-C14**.

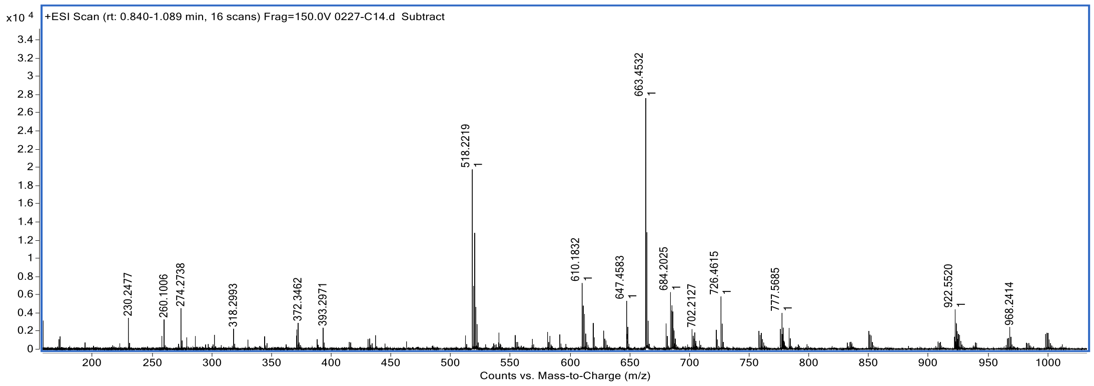

Figure S27. HR-ESI-MS spectrum of **DDAO-C14**.

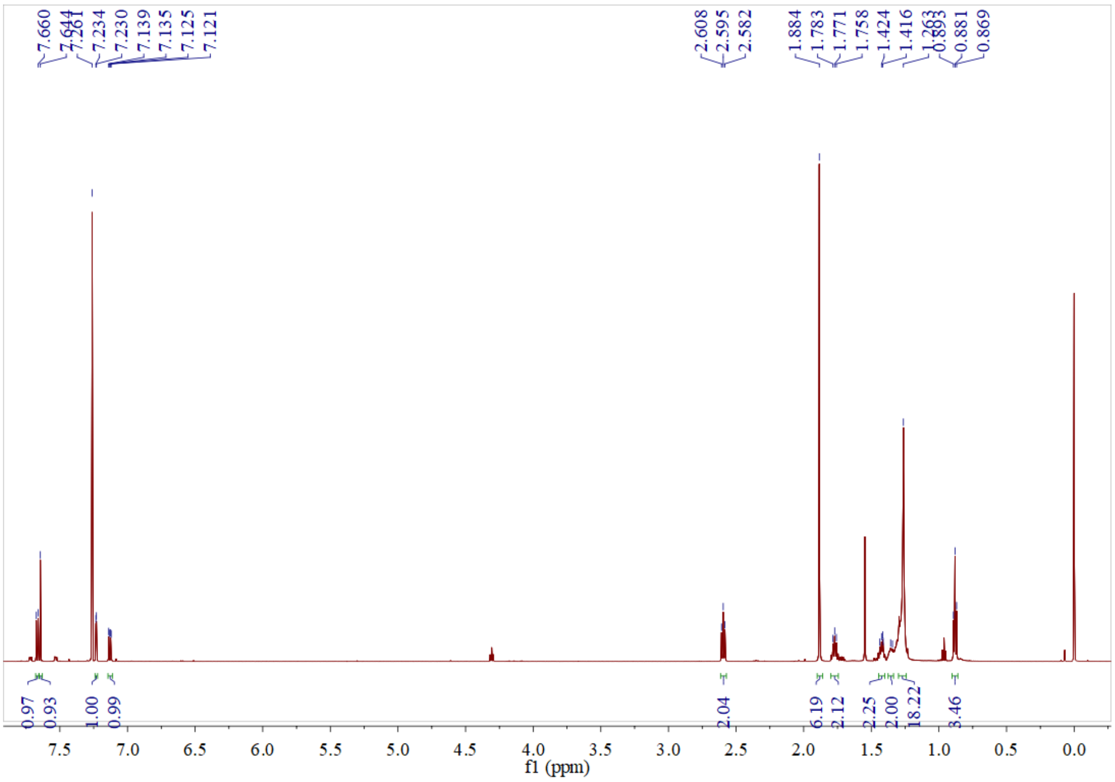

Figure S28. ^{1}H NMR spectrum of **DDAO-C15**.

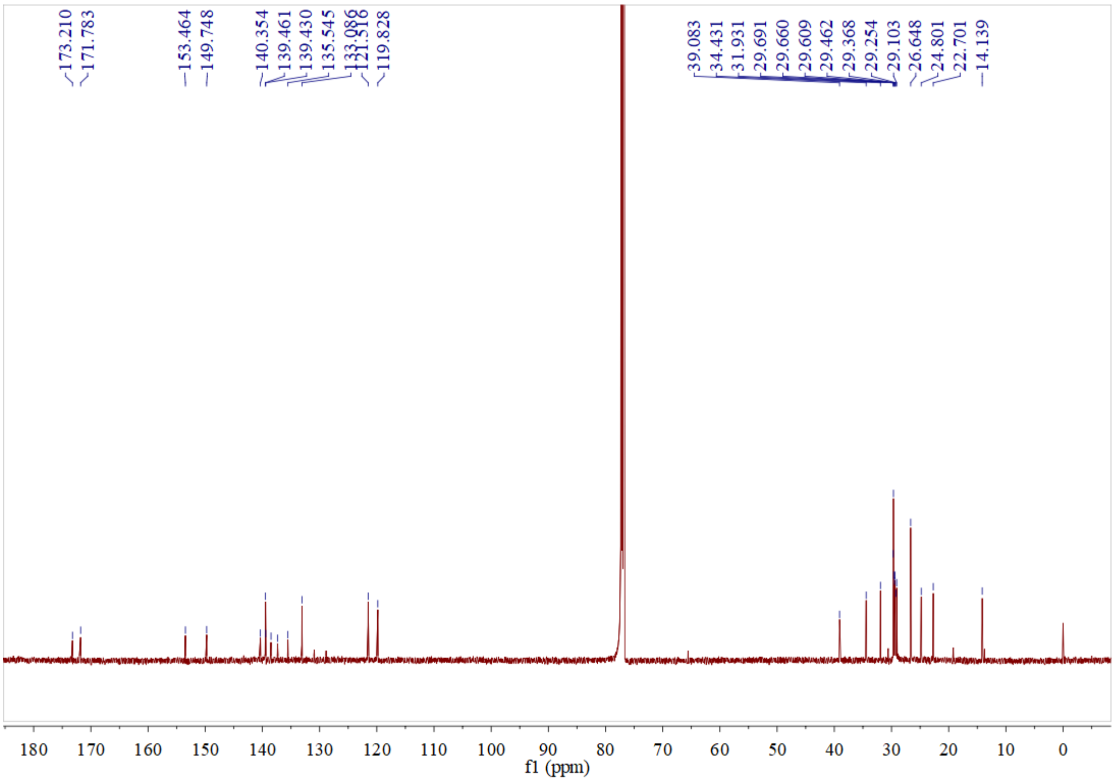

Figure S29. ^{13}C NMR spectrum of **DDAO-C15**.

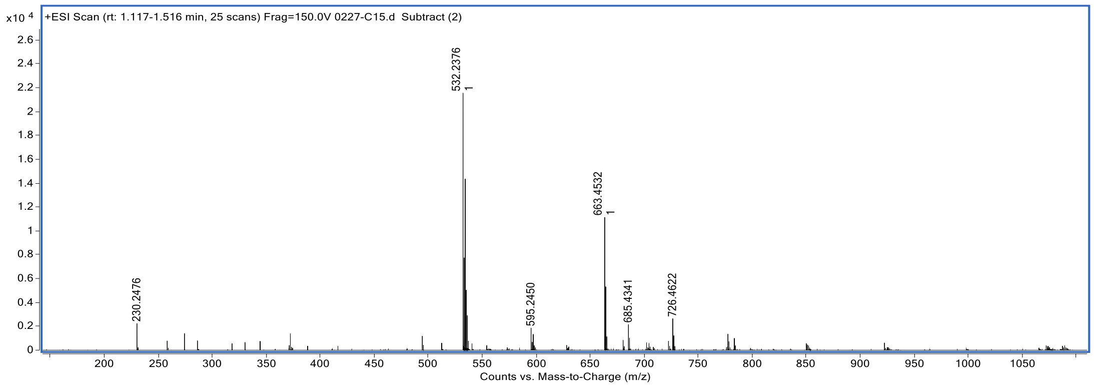

Figure S30. HR-ESI-MS of **DDAO-C15**.

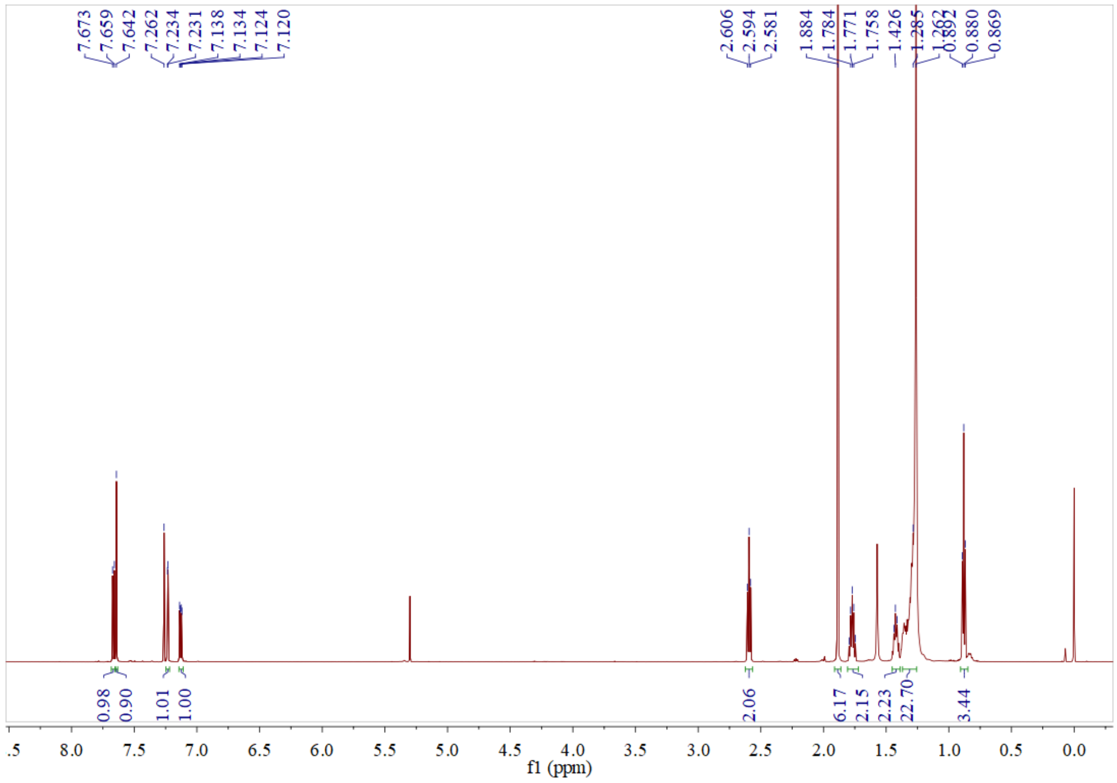

Figure S31. ^{1}H NMR spectrum of **DDAO-C16**.

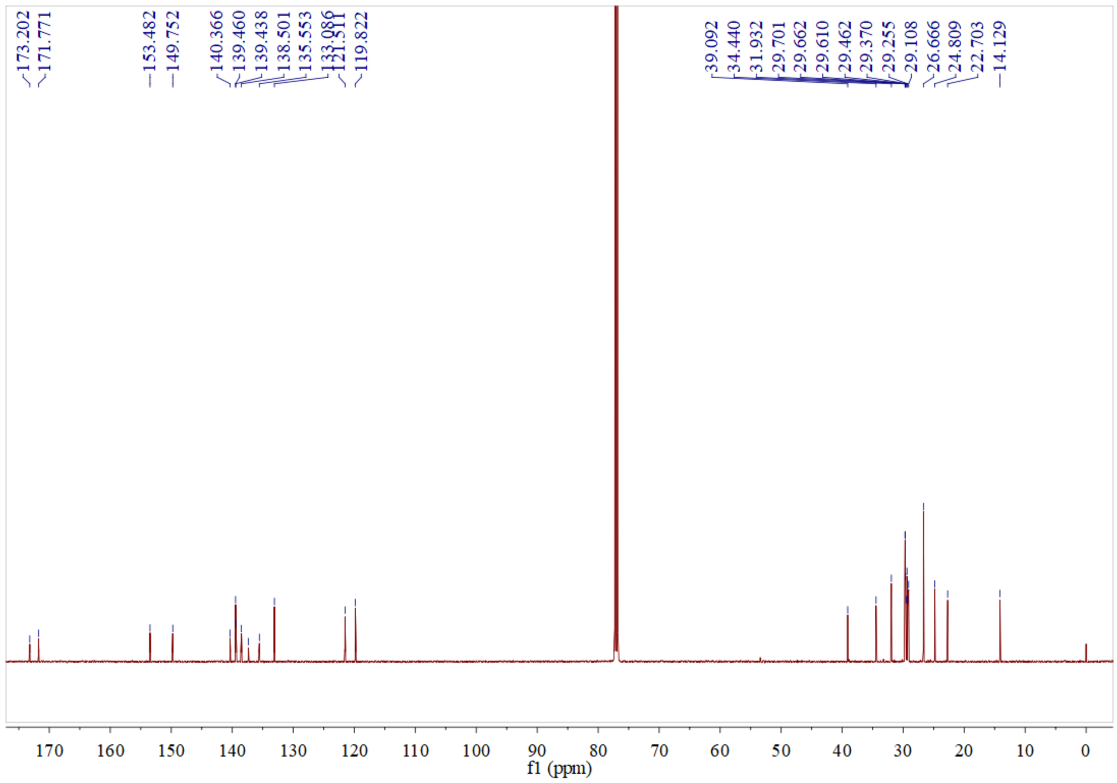

Figure S32. ^{13}C NMR spectrum of **DDAO-C16**.

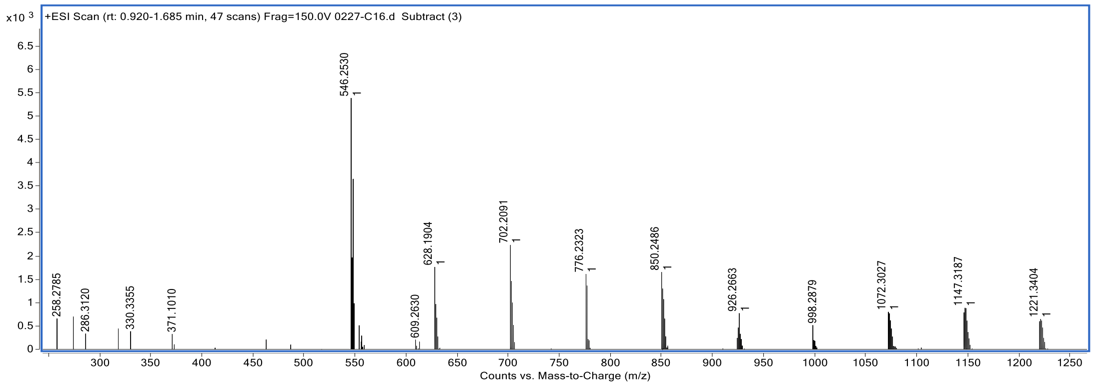

Figure S33. HR-ESI-MS spectrum of **DDAO-C16**.

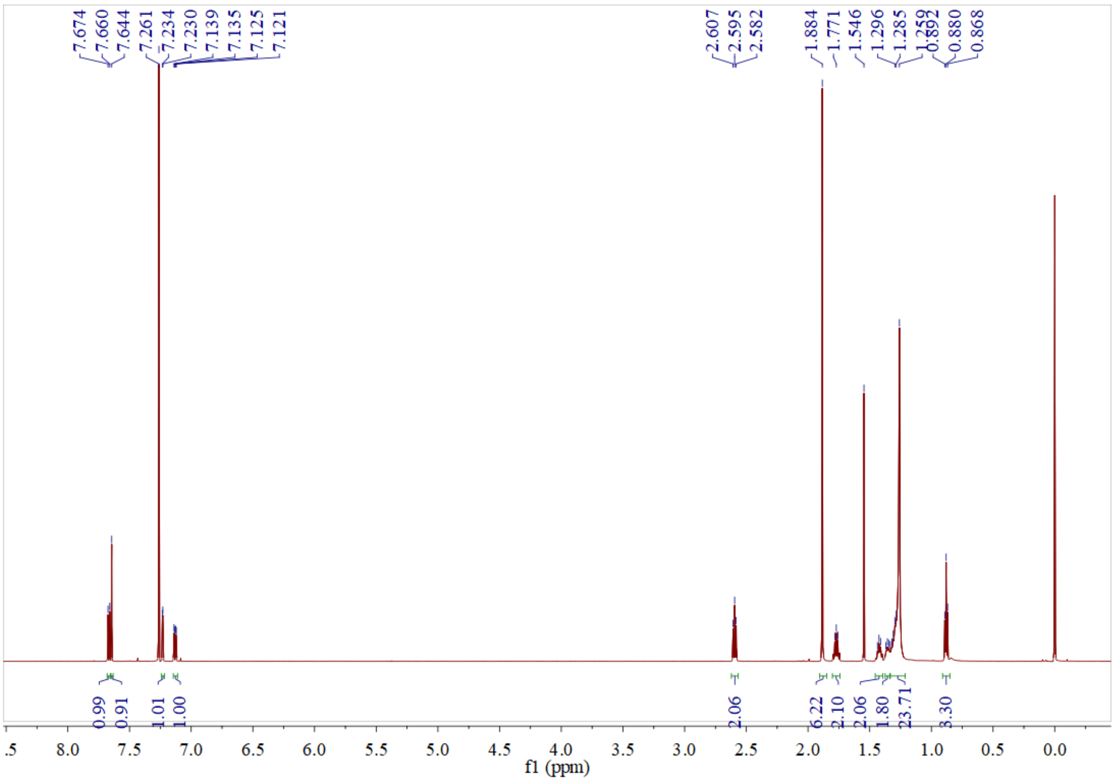

Figure S34. ^{1}H NMR spectrum of **DDAO-C18**.

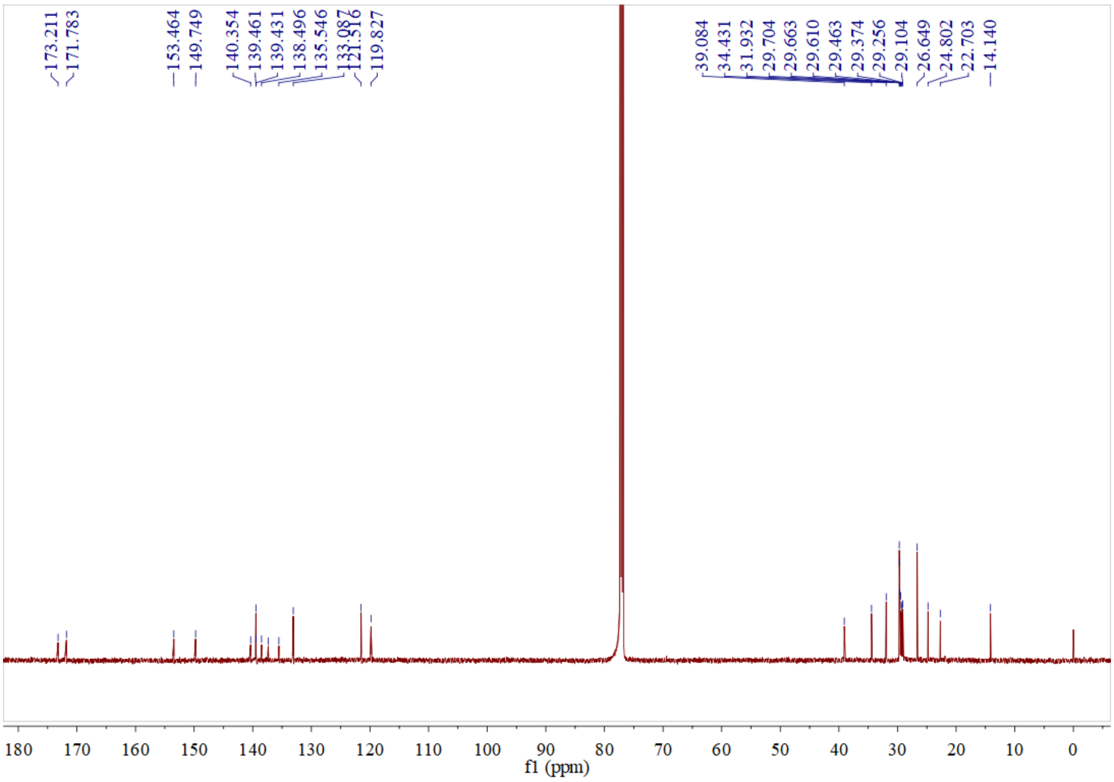

Figure S35. ^{13}C NMR spectrum of **DDAO-C18**.

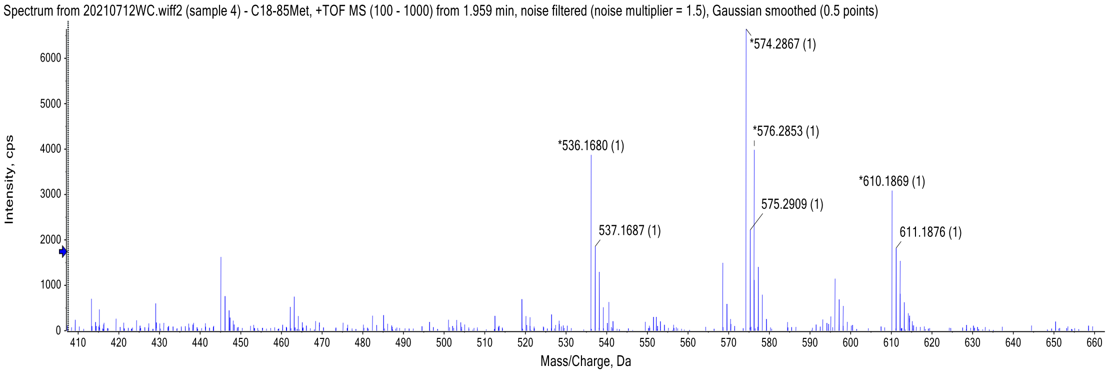

Figure S36. HR-ESI-MS spectrum of **DDAO-C18**.

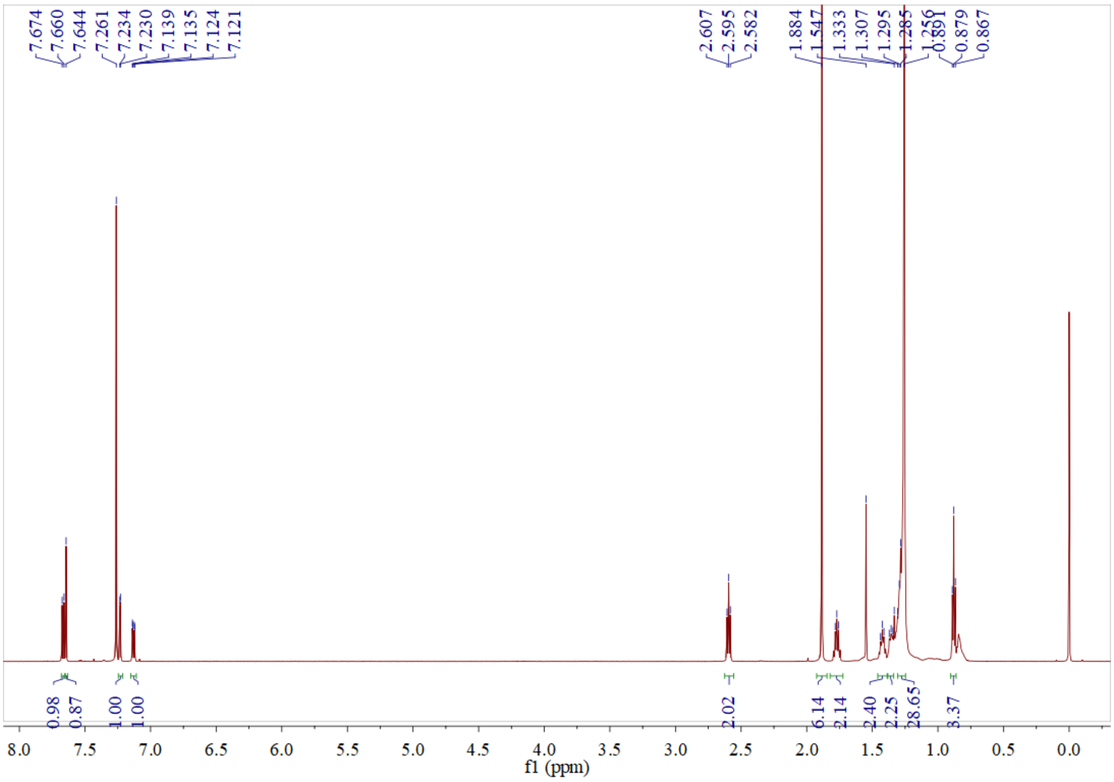

Figure S37. ^{1}H NMR spectrum of **DDAO-C20**.

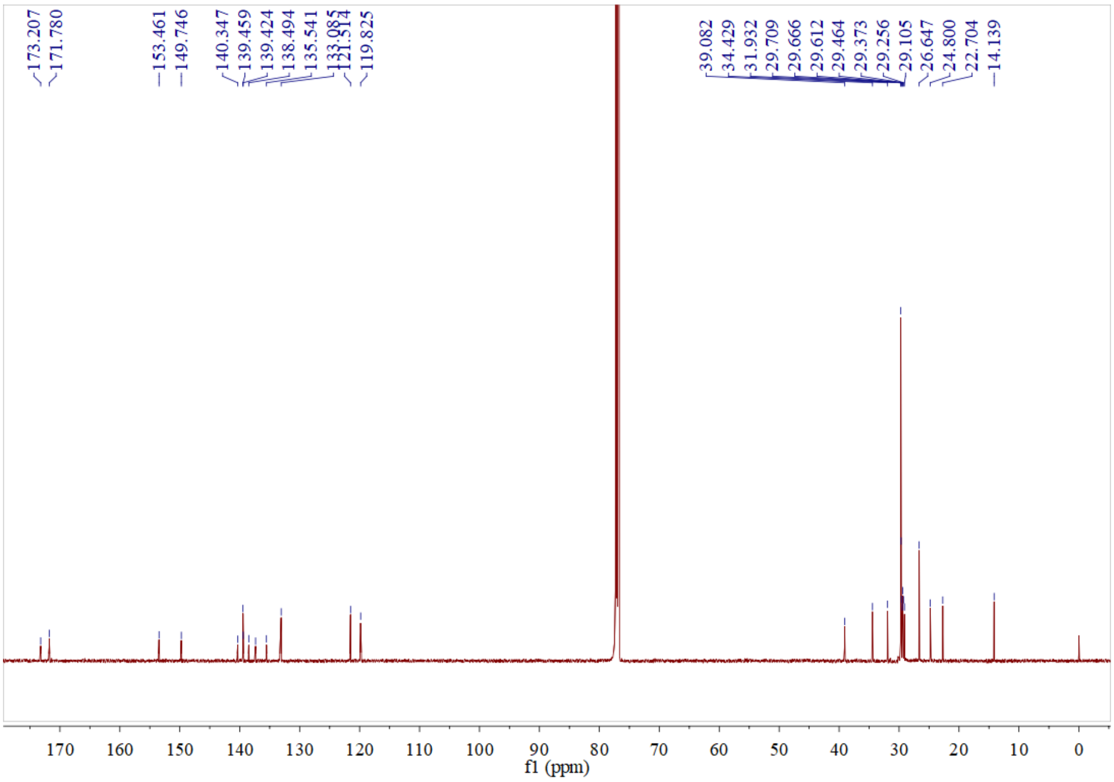

Figure S38. ^{13}C NMR spectrum of **DDAO-C20**.

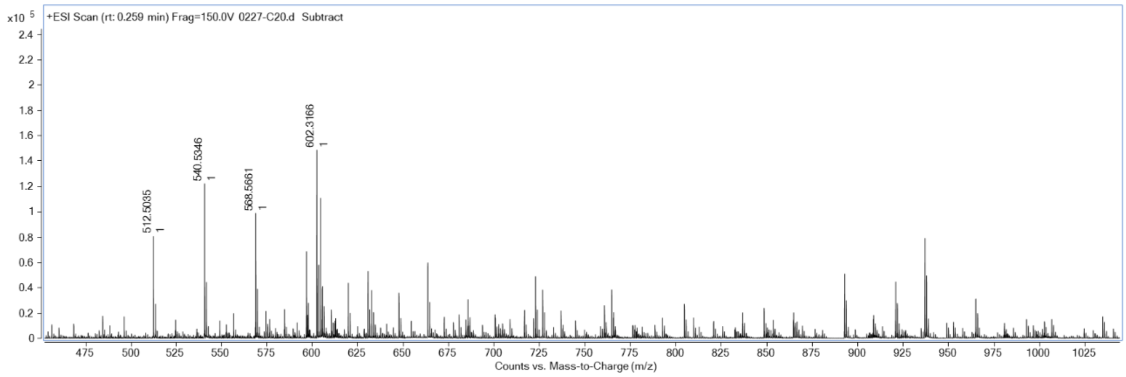

Figure S39. HR-ESI-MS spectrum of **DDAO-C20**.
